# Evaluation of the Sources, Precursors, and Processing of Aerosols at a High-Altitude Tropical Site

**DOI:** 10.1021/acsearthspacechem.2c00149

**Published:** 2022-09-12

**Authors:** Pamela A. Dominutti, Emmanuel Chevassus, Jean-Luc Baray, Jean-Luc Jaffrezo, Agnès Borbon, Aurèlie Colomb, Laurent Deguillaume, Samira El Gdachi, Stephan Houdier, Maud Leriche, Jean-Marc Metzger, Manon Rocco, Pierre Tulet, Karine Sellegri, Evelyn Freney

**Affiliations:** † Université Clermont-Auvergne, CNRS, UMR 6016, Laboratoire de Météorologie Physique (LaMP), Clermont-Ferrand 63000, France; ‡ Université Grenoble Alpes, UMR 5001, CNRS, IRD, Institut des Géosciences de l’Environnement (IGE), Grenoble 38400, France; § Laboratoire d’Aérologie (LAERO), UMR 5560, Toulouse 31400, France; ∥ Laboratoire de l’Atmosphère et des Cyclones (LACy), UMR 8105, Université de la Réunion, Saint-Denis de La Réunion 97744, France; ⊥ Centre pour l’étude et la simulation du climat à l’échelle régionale, Département des sciences de la terre et de l’atmosphère (ESCER), Université du Québec à Montréal, Montréal H2X 3Y7, Canada

**Keywords:** Secondary organic aerosols, ToF-ACSM, Positive matrix factorization, Cloud processing, Aqueous-phase chemistry, Southern Hemisphere

## Abstract

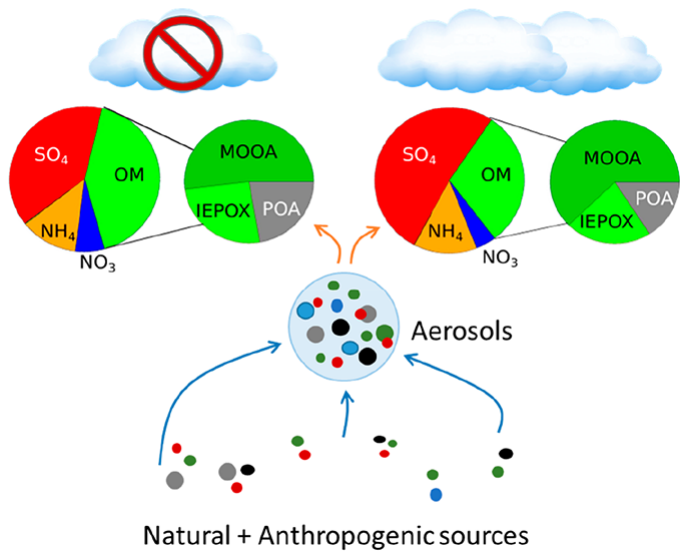

This work presents the results from a set of aerosol- and gas-phase measurements collected during the BIO-MAÏDO field campaign in Réunion between March 8 and April 5, 2019. Several offline and online sampling devices were installed at the Maïdo Observatory (MO), a remote high-altitude site in the Southern Hemisphere, allowing the physical and chemical characterization of atmospheric aerosols and gases. The evaluation of short-lived gas-phase measurements allows us to conclude that air masses sampled during this period contained little or no anthropogenic influence. The dominance of sulfate and organic species in the submicron fraction of the aerosol is similar to that measured at other coastal sites. Carboxylic acids on PM_10_ showed a significant contribution of oxalic acid, a typical tracer of aqueous processed air masses, increasing at the end of the campaign. This result agrees with the positive matrix factorization analysis of the submicron organic aerosol, where more oxidized organic aerosols (MOOAs) dominated the organic aerosol with an increasing contribution toward the end of the campaign. Using a combination of air mass trajectories (model predictions), it was possible to assess the impact of aqueous phase processing on the formation of secondary organic aerosols (SOAs). Our results show how specific chemical signatures and physical properties of air masses, possibly affected by cloud processing, can be identified at Réunion. These changes in properties are represented by a shift in aerosol size distribution to large diameters and an increased contribution of secondary sulfate and organic aerosols after cloud processing.

## 1 Introduction

Aerosols are an essential component of the Earth’s atmosphere. They have an impact on the radiative balance directly by scattering and absorbing solar radiation and indirectly by influencing cloud reflective properties (Twomey effect) as well as cloud lifetime and the formation of precipitation events (Albrecht effect) for liquid clouds. Over the past two decades, the increase in the number of aerosol chemistry studies by field measurements, both online and offline, has significantly improved our understanding of the composition and variability of aerosol chemistry. However, organic aerosol, which accounts for more than 50% of the submicron aerosols at continental sites, includes several thousand chemical compounds whose origins are not yet fully understood.^1−6^ In addition, these organic aerosols have been shown to play a crucial role in climate, as they can serve as cloud condensation nuclei^7−9^ and a source of ice-nucleating particles.^10^

Primary organic aerosols (POAs) arise from direct emissions whose sources are well identified in the literature, being derived from either anthropogenic or biogenic sources. POAs can include organic aerosols originating from natural sources such as spores, fungi, bacteria, viruses, and plant debris^11−15^ but are more often associated with anthropogenic sources such as combustion of fossil fuels or biofuels and open biomass burning.^16,17^ Secondary organic aerosols (SOAs) can be formed from the atmospheric oxidation of volatile organic compounds (VOCs)^18,19^ or originate from various processes such as heterogeneous reactions,^20^ photochemistry, and aqueous-phase oxidation.^21−23^ On the other hand, SOA formation processes are not as well understood, even though the global SOA contribution to organic aerosol (OA) emissions tends toward approximately 50% to 90%.^5^ Their complex chemical composition has been the subject of numerous studies over the two past decades. This, together with the evolution of online aerosol mass spectrometry, especially in the nonrefractory fraction of submicron aerosols (NR-PM_1_), has led to a significant increase in the characterization of secondary organic aerosol. Additionally, the application of receptor models such as Positive Matrix Factorization (PMF) allows the apportionment of the aerosols’ sources and their contribution to the emissions and formation processes in the atmosphere, especially for the organic fraction. However, these studies also highlight that the sources, formation mechanisms, and processes involved in the formation of SOA are still poorly understood.^24^ This is mainly due to measurement uncertainties and chemical complexities,^25^ where model predictions tend to systematically underestimate the observations.^2^ These differences between models and observations could be even more substantial at high altitudes.^24^ Moreover, recent studies have shown that the aqueous-phase processing of aerosols has a dominant impact on the formation of more oxidized SOA (more oxidized organic aerosol, MOOA). The contribution of MOOA to total organic aerosols increases as a function of relative humidity or liquid water content.^20,23,25−28^

Nevertheless, while the aerosol composition in the Northern Hemisphere has been extensively studied,^1,3,5^ the Southern Hemisphere faces a general lack of measurements. Moreover, the strong bias in global data coverage has already been highlighted in previous studies, limiting the assessment of the aerosols’ spatial distribution and characteristics over the globe.^29,30^ In this context, Réunion (21° S, 55° E), a volcanic tropical island located in the southwestern part of the Indian Ocean, is a particularly interesting place to study the physical and chemical processes involved in the formation of secondary aerosols. The tropical climate of the island favors the emission of biogenic volatile organic compounds (BVOCs) from vegetation,^31,32^ which in turn leads to high SOA formation rates.^33,34^ In addition, the particular location of Réunion is also under the influence of marine and anthropogenic sources (vehicular, shipping, and agricultural emissions), and it presents optimal conditions for transformations of chemical compounds, since it is a unique site for the study of SOA production. Moreover, frequently shallow convective clouds are formed and transported over the slopes of the Maïdo area, and air masses occasionally encounter these cloud events, which are generally dissipated before reaching the Maïdo Observatory.^35,36^ Therefore, air masses are often exposed to conditions that favor the aqueous-phase processing of secondary aerosols.

The objectives of this work were to evaluate a comprehensive set of gas and aerosol measurements obtained during the BIO-MAÏDO field campaign and to better understand the multiphase chemical mechanisms and biological properties of air masses (gas, aerosols, and clouds) and the mechanisms controlling the formation processes of secondary organic aerosol (SOA). Chemical and physical measurements were obtained at the equipped Maïdo Observatory (MO) site deployed at Réunion during March to April 2019. The analysis of aerosol chemical composition is addressed using offline filters (PM_10_) and online aerosol chemistry mass spectrometry measurements (NR-PM_1_). In addition, the aerosols’ size distribution is analyzed together with meteorological parameters and ancillary gas-phase measurements. Combined with high-resolution backward trajectories, these online measurements bring complementary insights into the complex atmospheric chemistry processes associated with the aerosol formation in tropical high-altitude marine sites and the largely uncharacterized Southern Hemisphere.

## 2 Materials and Methods

### 2.1 Sampling Strategy during the Field Campaign at MO

**Figure 1:**
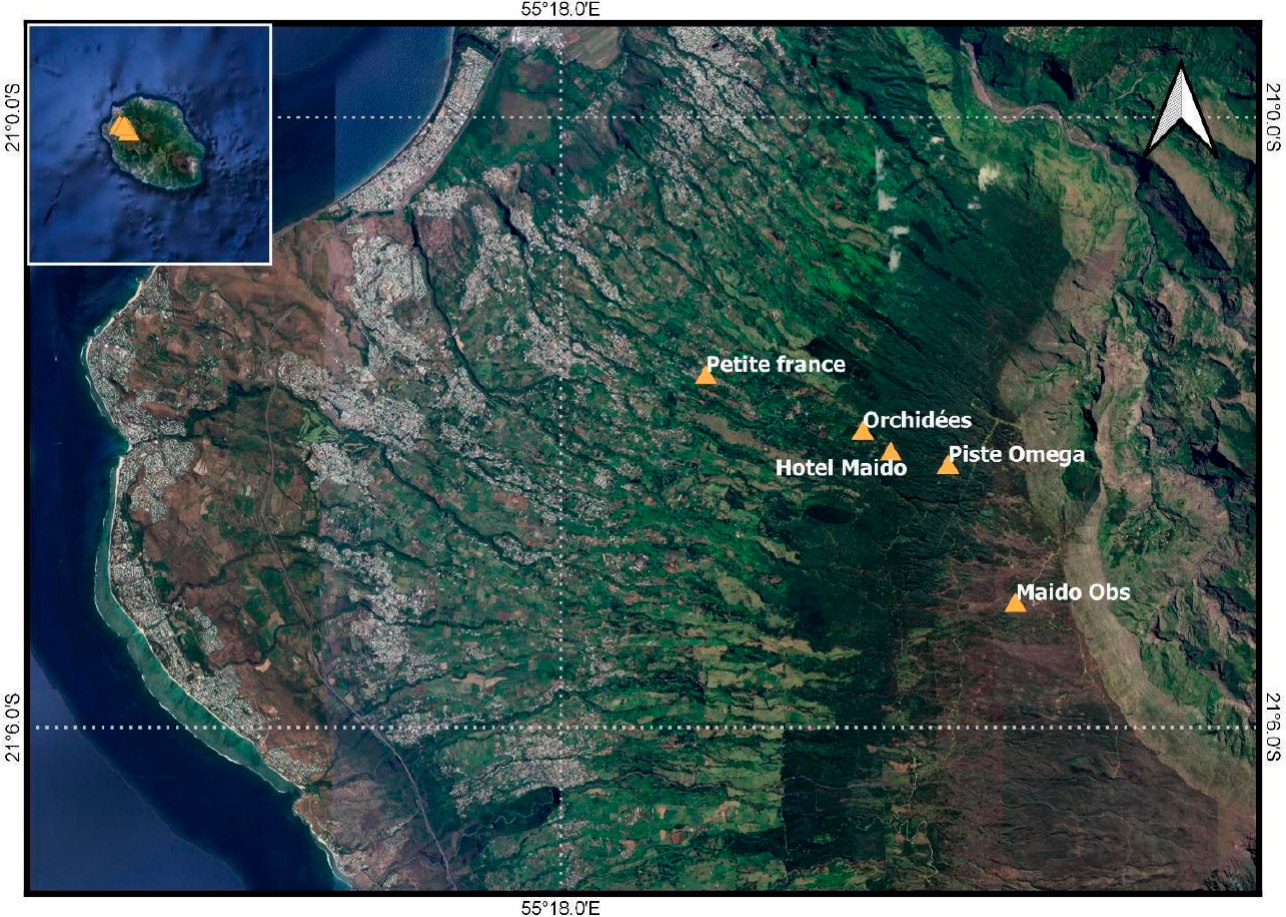
Location of the equipped sites deployed at Réunion during the BIO-MAÏDO field campaign. Aerosol measurements were developed at Maïdo Observatory and Petite France (965 m); cloud samples were obtained at Piste Omega (1760 m), and gas observations were obtained at Hotel Maïdo (1500 m), Petite France, and Maïdo Observatory (2160 m).

During March and April 2019, a series of instruments were installed at several key locations in Réunion (Figure 1) in the framework of the BIO-MAÏDO program. At the mountain-top Maïdo Observatory (MO) (2160 m.a.s.l.), the chemistry of submicron aerosols was analyzed online with a resolution of 10 min using a time-of-flight aerosol chemical speciation monitor (ToF-ACSM), providing information on the nonrefractory submicron fraction of organic and inorganic aerosols. In parallel, aerosols were sampled offline on filters for detailed aerosol chemistry using a high-volume sampler at the lower altitude station Petite France (PF; 965 m) and MO stations. High-time resolution (5 min) measurements of gas-phase volatile organic compounds (both biogenic and anthropogenic) were made using an online proton-transfer reaction mass spectrometer (PTR-MS) at both PF and MO stations.^31^ In this work, we only discuss the data collected at the MO site.

Cloud events were sampled at Piste Omega (PO), located on the ascending slope between PF and MO (Figure 1). A comprehensive chemical analysis was performed on collected cloudwater, evaluating their inorganic (ions, metals) and organic (amino acids, organic acids, sugars, dissolved organic matter, carbonyls, and low solubility volatile organic compounds) composition.^37^

The MO is situated close to the summit on the western part of the island at an altitude that allows for free troposphere (FT) chemical characterization at nighttime as well as boundary layer (BL) chemical characterization during daytime. Large-scale air mass dynamics influence the atmosphere at Réunion island, chiefly characterized by Hadley Cell-induced westerlies in the free troposphere^38^ and southeasterly trade winds near the ground. These flows are then separated into two flows as the trade winds glide along the island’s wake, resulting in northwesterly counterflows.^39,40^ During the night and early morning hours, katabatic winds and land-breeze prevail, and air mass arriving at Maïdo is separated from local and regional sources of pollution due to the strengthening of the large-scale subtropical subsidence. The site lies within the boundary layer from morning to late evening, characterized by adiabatic slope winds coupled with sea breeze.^38^ The site is impacted by primary sources from marine and biogenic vegetation origin as air masses pass through the lush forest covers, as reported during the FARCE campaign.^36^

### 2.2 The Maïdo Observatory (MO)

The MO is a fully equipped observatory described in Baray et al.^35^ It is part of the ACTRIS network (ACTRIS.eu, last accessed 8 March 2022) and has been labeled as a Global Atmospheric Watch (GAW) observation station since 2020 (Global Atmospheric Watch, https://public.wmo.int/en/programmes, last accessed 8 March 2022). The station is equipped with four whole air inlets, which allows the sampling of droplets during cloud events. In addition to the wide range of in situ aerosol measurements operating at the site (Sections 2.3 to 2.5), meteorological parameters, such as wind speed, wind direction, relative humidity (RH), temperature, and solar radiation, are measured using a Vaisala HMP45 automatic weather station (Figure S1).

Trace gases of interest include ozone (O_3_), nitric oxide (NO), nitrogen dioxide (NO_2_), and sulfur dioxide (SO_2_). Additionally, methane (CH_4_) and carbon monoxide (CO) mixing ratios were measured at the MO as part of the Integrated Carbon Observation System (ICOS; https://www.icos-cp.eu/, last accessed 8 March 2022) network. More details about measurement techniques and instruments can be found in Table S1 and on the OPAR Web site (https://opar.univ-reunion.fr/, last accessed 8 March 2022).

### 2.3 Aerosol Size Distributions

The particle size distribution (PSD) was measured using a custom-made differential mobility particle sizer (DMPS) with a condensation particle counter (CPC) of the type TSI, 3010. The DMPS provides the number of particles (cm^–3^) per particle diameter (*D*_p_) (expressed as d*N*/(d log *D*_p_)) every 8 min, where the diameters of the particles were separated into 14 different size classes from 13.7 to 650 nm.^38,41^ DMPS data were processed using the method described in Rose et al.^41^ and Wiedensohler et al.^42^ The characterization of aerosol modes was delimited to particles ranging from 13.7 to 90.7 nm for both Aitken and nucleation modes due to ambiguous boundaries between them and low bin number^43^ and 90 to 600 nm for accumulation mode.

### 2.4 Time of Flight Aerosol Chemical Speciation Monitor (ToF-ACSM)

Ambient nonrefractory submicron particles (NR-PM_1_) were monitored using a time-of-flight aerosol chemical speciation monitor (ToF-ACSM; Aerodyne Research Inc., USA) equipped with a standard vaporizer. The instrument provides a quantitative assessment of nonrefractory species, including both organic and inorganic species.^44^ At the MO, ambient air is sampled through a whole air inlet (WAI). The RH was continuously monitored in the sampling line to ensure that the RH never increased above 40% (average values were 32 ± 1%), excluding the need for additional drying devices. The sampled aerosol was then directed toward a critical orifice (130 μm) at a pressure of 2 Torr before passing through an aerodynamic lens^45^ toward a differentially pumped vacuum chamber where particles are flash vaporized on a heated tungsten surface (600 °C). The vaporized molecules are then ionized through electron impact ionization at 70 eV before being detected and quantified by the mass spectrometer as ion mass over charge (*m*/*z* 10–230) intensity ratios. The instrument alternates between sampling ambient air and sampling through a filter to subtract the signal due to air. The ToF-ACSM raw data were evaluated using the standard fragmentation table defined by Allan et al.,^46^ using the Igor-based (v6.37) software package TOFWARE version 2.5.13. The resolved mass concentrations include organics, nitrate (NO_3_^–^), sulfate (SO_4_^2–^), ammonium (NH_4_^+^), and chloride (Cl^–^).^44^ Instrument calibration was performed using 300 nm particles of ammonium nitrate and ammonium sulfate, giving a corresponding nitrate response factor (RF_NO3_) of 200 ions pg^–1^ and relative ionization efficiencies (RIE) of 3.12 for NH_4_ and 0.8 for SO_4_. Default RIE values were used for organic matter (1.4) and Cl (1.3).

The particle’s sampling efficiency in the ToF-ACSM varies with chemical composition, acidity, and aerosol water content. Therefore, overall particle losses were corrected using the composition-dependent collection efficiency (CDCE), which accounts for particle losses from particles bouncing on the heated filament surface as well as composition-dependent variations in the sampling efficiency^47^ (Section S1, Figure S2).

The intercomparison between ToF-ACSM, DMPS, and offline filters was performed to check the satisfactory metrological consistency over the whole campaign. DMPS aerosol size distribution measurements are converted to mass concentrations and compared with ToF-ACSM mass concentrations (Section S2, Figure S3). A robust mass closure between ToF-ACSM and DMPS was achieved (*R* = 0.9, slope = 1.07), confirming that the ToF-ACSM accurately represents the submicron aerosol and refractory species (mineral dust, sea salt, soot) had low to negligible contributions to the submicron aerosol throughout the campaign. More details about DMPS can be found in Section S2, Figure S4.

### 2.5 Offline Filter Sampling and Analyses

A total of 32 pure quartz fiber filters were collected over the campaign period for an overall duration of 10–12 h each during the night (9 p.m. to 8 a.m. local time (LT)) and daytime (from 8 a.m. to 9 p.m. LT). Filters were sampled from March 8 to April 4 using a PM_10_ high-volume impact sampler (Digitel DA80^48^). Prior to use, the filters were heated to 500 °C for 12 h to avoid organic contamination. Blank filters were collected in the field to determine the detection limits (DLs) and verify that no contamination was present during the transport, setup, and recovery of the samples. After collection, filter samples were wrapped in aluminum foil, sealed in plastic bags, and stored at <4 °C until further chemical analysis. Anions (Cl^–^, NO_3_^–^, SO_4_^2–^), cations (NH_4_^+^, Ca^2+^, Na^+^, Mg^2+^, K^+^), and light organic acids (methanesulfonic acid and oxalic acid) were analyzed by ionic chromatography.^49^ In addition, carbonaceous aerosols were measured using a sunset lab analyzer following the EUSAAR-2 thermal protocol.^50^ These measurements included elemental carbon (EC), or soot, originating from the incomplete combustion of various organic materials, and organic carbon (OC). Sugars (levoglucosan, mannosan, galactosan, rhamnose, trehalose, and glucose) and polyols (inositol, glycerol, erythritol, xylitol, arabitol, sorbitol, and mannitol) were quantified using high-performance liquid chromatography with a pulsed Amperometric detector (HPLC-PAD^51^). A total of 16 dicarboxylic acids were also measured using high-performance liquid chromatography–mass spectrometry.^52^ The analytical system was composed of an autosampler from Dionex coupled with a degassing system from Alltech, a thermostatic column compartment, a GP40 quaternary pump (Dionex), and a triple multipol-3D ion trap mass spectrometer (LCQ-FLEET from Thermo-Fisher) equipped with an electrospray ionization source (ESI). A central supply of high purity nitrogen was used as a nebulizer and drier gas for the ESI source. Thus, the ESI-MS source was set in negative polarity with a capillary temperature of 350 °C and spray voltage of 5 kV. Proper separation was achieved with a Synergi 4 μm Fusion-RP 80A column (250 × 3 mm ID, 4 μm particle size, from Phenomenex) with an eluent flow rate of 0.5 mL.min^–1^ and a gradient of water/acetonitrile.

### 2.6 Proton-Transfer Reaction Mass Spectrometer (PTR-MS)

Volatile organic compounds (VOCs) were measured at the MO using a quadrupole-based proton-transfer reaction mass spectrometer (PTR-MS; Ionicon Analytik GmbH, Austria) with a high sensitivity (10–100 pptv) and fast response time (∼100 × 10^–3^ s).^32^ Briefly, ambient VOCs are ionized by proton transfer from hollow cathode discharge reactant H_3_O^+^ (H_3_O^+^ + R → RH^+^ + H_2_O), then pumped through a drift tube reactor, and detected by a quadrupole mass spectrometer. The instrument was calibrated using VOCs standard samples, and full details can be found in Verreyken et al.^32^ and Rocco et al.^31^ The species of interest included methanol (*m*/*z* 33), acetonitrile (*m*/*z* 42), acetaldehyde (*m*/*z* 45), acetic acid (*m*/*z* 61), acetone (*m*/*z* 59), dimethyl sulfide (DMS; *m*/*z* 63), isoprene (*m*/*z* 69), methyl vinyl ketone + methacrolein + isoprene hydroxy hydroperoxides (MVK+MACR+ISOPOOH) (*m*/*z* 71), methyl ethyl ketone (MEK; *m*/*z* 73), benzene (*m*/*z* 79), toluene (*m*/*z* 93), xylenes (*m*/*z* 107), and monoterpenes (*m*/*z* 137 and *m*/*z* 81).

### 2.7 Data Analysis

#### 2.7.1 Positive Matrix Factorization (PMF)

Individual mass spectra (MS) were analyzed using the Positive Matrix Factorization (PMF^55^) model to deconvolve the ToF-ACSM organic contribution to NR-PM_1_. PMF is a source-receptor model widely used to identify and quantify source contributions to samples, study primary or secondary sources, or study the chemical and dynamical influences on the sources.^56^

PMF aims to solve a matrix equation using a weighted least-squares approach,
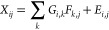
1where *i* represents the time index, *j* represents the species, and *k* is the factor number. *X*_*i,j*_ is the matrix of our mass spectra; *G*_*i,k*_ is the matrix of the factor time series. *F*_*k,j*_ is the matrix of the factor profiles, and *E*_*i,j*_ is the model residual matrix (difference between the data matrix and the fitted solution). The input error matrix *S*_*i,j*_ includes the measurement uncertainty (ion-counting statistics and ion-to-ion noise ratio) as well as the blank variability. *G* and *F* values are iteratively fitted to the data using a least-squares gradient descent algorithm minimizing a fit quality parameter *Q*, defined as
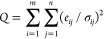
2where σ_*ij*_ is the standard deviation of the estimated errors matrix from the matrix *X*.

The IGOR PRO Source Finder (SoFi v6.8.1) toolkit^57^ was used to run the PMF algorithm, which provides a priori factor profiles by testing the different rotational techniques available in the ME-2 engine.^58^ Source profiles were assessed using the unconstrained factors rotational Fpeak tool. Chemical species with a signal-to-noise ratio (S/N) below 0.2 and above 10 were automatically excluded from the analysis. In addition, the ACSM signal for air (N_2_^+^ at *m*/*z* 29) was removed, as this signal is often associated with background noise.^59^ Time series and matrix size, respectively, consisted of 9071 and 124 points. Solutions were explored for Fpeaks (rotations) between −1 and 1 with 0.1 steps. The rotational freedom of the chosen solution was confirmed by the range of *Q*/*Q*_exp_ ratio values, all at least 3% above the minimum *Q*/*Q*_exp_ ratio (as recommended by Zhang et al.^60^). PMF was run with solutions (sources) ranging from 2 to 6 factors (Section S3). The final solution of 3 factors was chosen on the basis of optimal *Q*/*Q*_exp_ values, physically meaningful reference profiles, and time series (Figures S5 and S6) and their correlations with external factors.

The total factor number was assessed by the visual inspection of *Q*/*Q*_exp_ ratios, correlations of factor MS profiles, and factor time series with external factors. The resolved PMF factors were compared to reference mass spectra listed on the aerosol mass spectrometer database (http://cires1.colorado.edu/jimenez-group/AMSsd/, last accessed 8 March 2022): isoprene epoxydiols organic aerosols (IEPOXOAs),^61^ aqueous phase processing-related more oxidized organic aerosols (MOOAs),^62^ and oxalic acid and malonic acid^63^ as well as representative POA signatures.^62,64^ Oleic acid and palmitic acid reference mass spectra were used to check for a primary aliphatic-rich organic aerosol presence. Amino acid spectra were also checked, which may indicate the aerosols’ age and origin.^65,66^

The factor’s solutions were also verified regarding their a posteriori correlations with ancillary meteorological parameters, aerosol size distribution modes (nucleation and accumulation modes), reactive gases, and VOC mixing ratios as well as the time series of NR-PM_1_ inorganic aerosol mass concentrations.

#### 2.7.2 High-Resolution Trajectories

Forward and backward spatial–temporal high-resolution local atmospheric trajectories were calculated to analyze the dynamical context of the gas, aerosol, and cloudwater measurements performed at the different sites of the BIO-MAÏDO campaign. The model used is Meso-CAT. This model results from the combination of the CAT trajectory code for the advection calculation^67^ and a nonhydrostatic mesoscale atmospheric model, Meso-NH^68^ (http://mesonh.aero.obs-mip.fr, last accessed 12 May 2022), to provide the dynamical fields (wind, temperature, and humidity) and topography at high spatial (grid nested domains at 100 and 500 m on 72 vertical levels) and temporal resolutions (1 h). The vertical resolution decreases with height from less than 10 m at ground level to up to 1 km to the top of the domain at 25 km.

The trajectory configuration is as follows: a cluster of 75 trajectories regularly distributed in a parallelogram of 500 m width and 50 m height centered on the measurement site; each trajectory was calculated for a maximum total duration of 12 h with a temporal resolution of 5 min. Rocco et al.^31^ showed that this configuration could highlight small-scale atmospheric circulations such as land, sea, and slope breezes that influence measurements of short-lived species at Réunion and provide information about the connections between MO and the other sampling sites (Petite France, Piste Omega). Additionally, modeled water vapor and cloud mixing ratios (in g kg^–1^), provided by Meso-NH high-resolution simulations, have been interpolated on all Meso-CAT trajectory points to provide information on the cloud conditions encountered during air masses trajectories.

## 3 Results and Discussion

### 3.1 Meteorological Conditions and Gas-Phase Measurements

Figure S1 presents the typical diurnal profiles of meteorological parameters for the entire campaign at the MO. During the BIO-MAÏDO campaign, the dominant origin of air masses at the MO was south/southwesterly during the daytime and north/northeasterly during the nighttime period. Relative humidity (RH) varied between 60% and 80% with the highest values observed in the afternoon (from 18 LT). Air temperature presented the maximum values in the middle of the morning (10 a.m. LT) and a decreasing trend for the day with minimum values at night (3 a.m. LT).

Gas-phase measurements of CO, NO, and SO_2_ showed overall low concentrations (hourly average below 30, 0.06, and 0.19 ppb, respectively), ruling out any significant local anthropogenic or volcanic sources. In general, when the site is influenced by volcanic activity from the nearby volcano (Piton de la Fournaise), the SO_2_ concentrations increase to between 100 and 300 ppbv.^41^ VOC measurements confirmed this with measurements of aromatic hydrocarbons (markers for anthropogenic activities^69^), such as xylene (0.012 ± 0.040 ppbv), benzene (0.010 ± 0.027 ppbv), and toluene (below the detection limit), all being close to the detection limit of the instrument, and their values are considered to represent clean background conditions.^69^ For example, average benzene and toluene mixing ratio values reported at other remote mountain sites are 0.32 and 0.20 ppbv, respectively, for Puy de Dôme, France, and 0.06 and 0.02 ppbv, respectively, for Hohenpeißenberg.^70^ However, biogenic species, such as isoprene, dominated the measured VOCs. The isoprene measured concentrations (0.1300 ± 0.0005 ppbv) could be emitted by both endemic vegetation (*Erica arborescens* and *reunionensis* and *Acacia heterophylla*(36)) and marine phytoplankton.^71^ Conversely, monoterpenes, which can be emitted by coniferous trees (such as *Cryptomeria japonica*(36)), did not present high concentrations during our study (0.0110 ± 0.0008 ppbv). High concentrations of intermediate photochemical isoprene oxidation byproducts,^72^ MVK/MACR/ISOPOOH (0.0800 ± 0.0029 ppbv), highlight the contribution of biogenic sources over the island. Isoprene photooxidation is a very important driver of atmospheric chemistry over forested regions. Isoprene reacts with hydroxyl radicals (OH) and molecular oxygen to produce isoprene peroxy radicals (ISOPOO). These radicals react with hydroperoxyl radicals (HO_2_) to produce hydroxyhydroperoxides (ISOPOOH) or react with nitric oxide (NO) to produce methyl vinyl ketone (MVK) and methacrolein (MACR). The quadrupole detector used in La Réunion cannot distinguish between the isobaric molecules MVK and MACR, and its decomposition interferes with ISOPOOH. As a consequence, we report the data at *m*/*z* 71 as the sum of the three isomers (MACR+MVK+ISOPOOH), even though it was only calibrated for MVK+MACR.^125,126^

Finally, the mean MVK+MACR+ISOPOOH mass concentrations over the isoprene ratio was 1.35. This ratio is considerably higher than those measured in tropical rainforests (0.16 and 0.22),^73^ suggesting that the air masses were chemically aged and contained higher concentrations of more oxidized species. VOC observations highlight the high formaldehyde mixing ratio (1.280 ± 0.017 ppbv), which also suggests rapid oxidation of isoprene (first-generation oxidation byproduct^74^). Following isoprene, high concentrations of oxygenated compounds such as methanol (1.020 ± 0.016 ppbv), acetone (0.440 ± 0.004 ppbv), acetic acid (0.180 ± 0.052 ppbv), methyl ethyl ketone (MEK; 0.047 ± 0.001 ppbv), and acetaldehyde (0.170 ± 0.004 ppbv) were measured, all of which have been identified in the literature as BVOC released by vegetation and oceans^75−77^ or associated with both biogenic and anthropogenic sources in the case of acetaldehyde^78,79^ and MEK.^80^ Acetonitrile, which is considered a tracer for biomass burning,^81^ and dimethyl sulfide (DMS), emitted by phytoplankton,^71^ were both below the detection limit. Globally, VOC measurements were dominated by the presence of biogenic species from vegetation and oxygenated compounds. Our results indicate the presence of aged air masses at the MO and the low concentrations of primary anthropogenic VOCs during the field campaign.

### 3.2 Chemical Composition of PM_10_ Measurements

**Figure 2:**
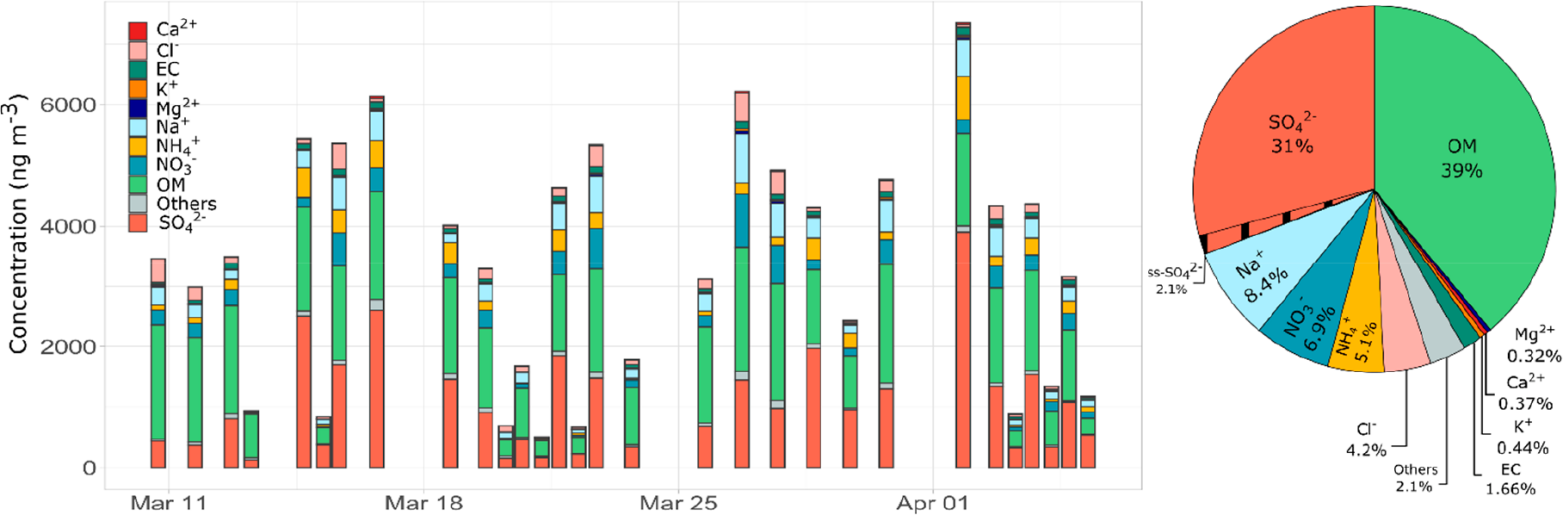
PM_10_ composition time series of organic and inorganic matter obtained in filters and the PM_10_ relative contribution over the whole campaign.

Offline PM_10_ filter measurements show that organic matter (OM) was the dominant fraction (39%, on average) of the aerosol composition followed by SO_4_^2–^ (31%), Na^+^ (8.4%), NO_3_^–^ (6.9%), NH_4_^+^ (5.1%), Cl^–^ (4.2%), K^+^ (0.44%), Mg^2+^ (0.32%), and Ca^2+^ (0.37%) (Figure 2). This composition is similar to long-term observations of PM_10_ obtained at the MO.^83^ In order to assess the impact of the marine sources, it was first assumed that NaCl interacts with acidic species (such as HNO_3_ or H_2_SO_4_), resulting in the formation of gas-phase NH_4_Cl or HCl, leaving the aerosols enriched in nitrate and nonsea salt (*nss*) sulfate and depleted in Cl^–^.^84,85^ The deposition of HCl gas may cause another possible loss mechanism of chlorine to the sea surface.^86^ The Cl^–^/Na^+^ average ratio of our measurements was 0.5 and ranged from 0.11 to 1.31; the reference seawater ratios ranged from 1.8^87^ to 1.9,^88^ but more recent studies in mesocosm systems detected ratios of 1.2 ± 0.12.^89^ This result suggests possible chloride depletion occurred between emission and sampling at the MO, implying that fresh sea salt emissions did not strongly impact the site. In such conditions and when it is assumed that Na^+^ solely comes from sea salt, the coefficients from Terzi et al.^90^ are then used to determine the *nss* fractions, selecting Na^+^ as a reference tracer.

Ca^2+^, Mg^2+^, and Cl^–^ show a prevailing marine origin (*nss* fractions are below the detection limits), while *nss*-SO_4_^2–^ largely contributes (97%) to the measured sulfate. *nss*-SO_4_^2–^ can be derived from dimethyl sulfide (DMS) produced either by biological ocean activity or from continental sources of sulfate. Methanesulfonic acid (MSA), a rather stable byproduct of DMS oxidation by OH radicals, is often used to estimate marine biogenic contribution.^91^ As such, the MSA to *nss*-SO_4_^2–^ (MSA/*nss*-SO_4_^2–^) ratio provides an indication of the relative contributions of DMS and anthropogenic sources to total sulfate levels. A high ratio suggests that a considerable fraction of the total *nss*-SO_4_^2–^ burden being observed is derived from the atmospheric oxidation of DMS, while a low ratio implies that the contribution of DMS to the total *nss*-SO_4_^2–^ burden being observed is low.^92^ This ratio has long been measured in several parts of the globe. The MSA/*nss*-SO_4_^2–^ ratio previously observed in subtropical and tropical oceanic areas varies between 0.005 and 0.070.^92−95^ Our result shows that the MSA/*nss*-SO_4_^2–^ ratio measured at the MO (0.0007) is 6 to 40 times lower than that observed in other regions. The MSA average concentrations observed in our study (0.78 ng m^–3^) are much lower than those reported in a recent study at Réunion by factors of 10 to 20 (11.7–8.3 ng m^–3^).^83^ These differences could be related to the several days under the detection limit concentrations during our field campaign. In addition, both studies were performed in different periods of the year, probably affecting the seasonality of the MSA’s emission processes to a different extent.

In addition, the anthropogenic contribution of *nss*-SO_4_^2–^ was assessed using the NO_3_^–^/*nss*-SO_4_^2–^ ratio, which gave an average value of 0.28, typical for remote areas with low anthropogenic pollution.^96^ The low contribution of anthropogenic sources is also confirmed by the low contribution of K^+^ to water-soluble ions (1%), confirming negligible biomass burning emissions, and also by the total organic carbon levels being 20 times lower than typical urban aerosols (ref (97) and the references therein). Substantial differences were observed in the total PM_10_ mass concentrations between the day (8 a.m to 9 p.m. LT) and the night (9 p.m to 8 a.m. LT) filters (by a factor of 4, Figure S7) with higher concentrations collected during the daytime (by a factor of 4). These differences, also previously observed,^83^ are related to the altitude of the MO, which is under boundary layer conditions during daytime and frequently under free troposphere conditions at nighttime. Despite the diurnal differences in the mass concentrations, the contributions of different chemical species did not vary significantly; a decrease was observed in both NO_3_ and NH_4_, and there was an increase in the contribution of OM from 36.4% up to 40.3% (Figure S7). However, it should be noted that overall mass concentrations measured at night are less than 0.6 μg m^–3^.

Filters were analyzed for the presence of sugars and polyols (sugar alcohols) and several carboxylic acids. The linear regression analyses between arabitol and mannitol (*R*^2^ = 0.99, slope = 0.64) and between glucose and polyols (*R*^2^ = 0.99, slope = 0.8) present strong correlations, suggesting similar emission sources. These sugar species could be emitted through resuspension of surface soils and associated bacterial/fungal spores (containing polyols and primary sugar compounds) or via a direct input resulting from microbial activities.^98^ The arabitol-over-mannitol ratio is much lower than the values reported in previous studies during summer maximums.^98^ Sugar alcohols present a seasonal profile in the Northern Hemisphere with maximum values obtained in summer and minimum values, in winter. The differences observed with our MO results could be related to our measurements being performed in the transition between summer and winter seasons in high-altitude sites. We analyzed the relative distribution of glucose (a marker for plant materials or soil emissions) and sugar alcohols (tracers of airborne fungi and bacteria^99^). Glucose was the dominant sugar (47%) at MO, followed by mannitol (21%), arabitol (15%), trehalose (8%), erythritol (6%), and other species (glycerol, inositol, sorbitol). Glucose, arabitol, and mannitol contributions are in line with the values reported in the literature,^98^ suggesting the contribution of similar emission sources. However, sugars and polyol tracers only contributed 1.3% on average to the total OM measured, which is lower than that observed in continental studies where the annual average contribution can reach up to 40% on some specific days.^98^ The concentrations of biogenic species (such as sugars alcohols) follow, globally, a seasonal cycle. However, this cycle has not yet been reported in Réunion; it is then difficult to identify if our field measurements were under a strong or weak emission period. Our results indicate that more investigation and observations are needed to better understand the seasonality, biome characteristics, sources, and formation processes of sugars in the island.

Sixteen carboxylic acids were quantified from the filter samples, contributing on average from 0.14% to 4% of the total organic matter. The temporal evolution of OM concentrations shows a dissimilar profile during the field campaign with two distinct periods. The first period is from March 8 to 21 (Period 1), during which only 1% of the OM could be characterized, and the second period (Period 2) is from March 22 to April 4, where up to 5% of the OM was revealed.

The second period is characterized by a prevalence of short-chain carboxylic acids (Figure S8), namely, oxalic acid, followed by malonic and succinic acids. These observations are similar to those of Wang et al.^100^ and Kawamura and Sakaguchi,^97^ who reported preferential production and accumulation of oxalic acid in the remote tropical Pacific Ocean linked to significant in-cloud production of both secondary sulfate and oxalic acid. The linear regression slope between oxalic acid and *nss*-SO_4_^2–^ for the whole campaign (*R* = 0.36, slope = 0.037) is in line with the slope values reported over East Asia coastal and nearby coastal sites by Bikkina et al.^101^ (Bay of Bengal, 0.019; Hong Kong, 0.034; Guangzhou, 0.033; Nanjing, 0.029; ACE-ASIA, 0.050). However, the low correlation between these two species suggests that other chemical processes also played an important role, such as the photochemical oxidation of organic precursors. Interestingly, the correlation coefficient substantially changes for the observations obtained at the end of Period 2 (*R* = 0.86, slope = 0.047), suggesting a higher impact on the in-cloud production of both compounds.

In terms of meteorological conditions, the comparison between the two sampling periods does not depict substantial differences in the diurnal temperature profiles (Figure S9). Nonetheless, the solar flux maxima observed during the first period, reaching up to 800 W m^–2^, was higher than that observed in the second period (up to 650 W m^–2^). In contrast, slightly higher RH values were observed in the second period. These differences could be related to the presence of cloud events, which were more intense during Period 2 (Figure S9).^37^

**Figure 3:**
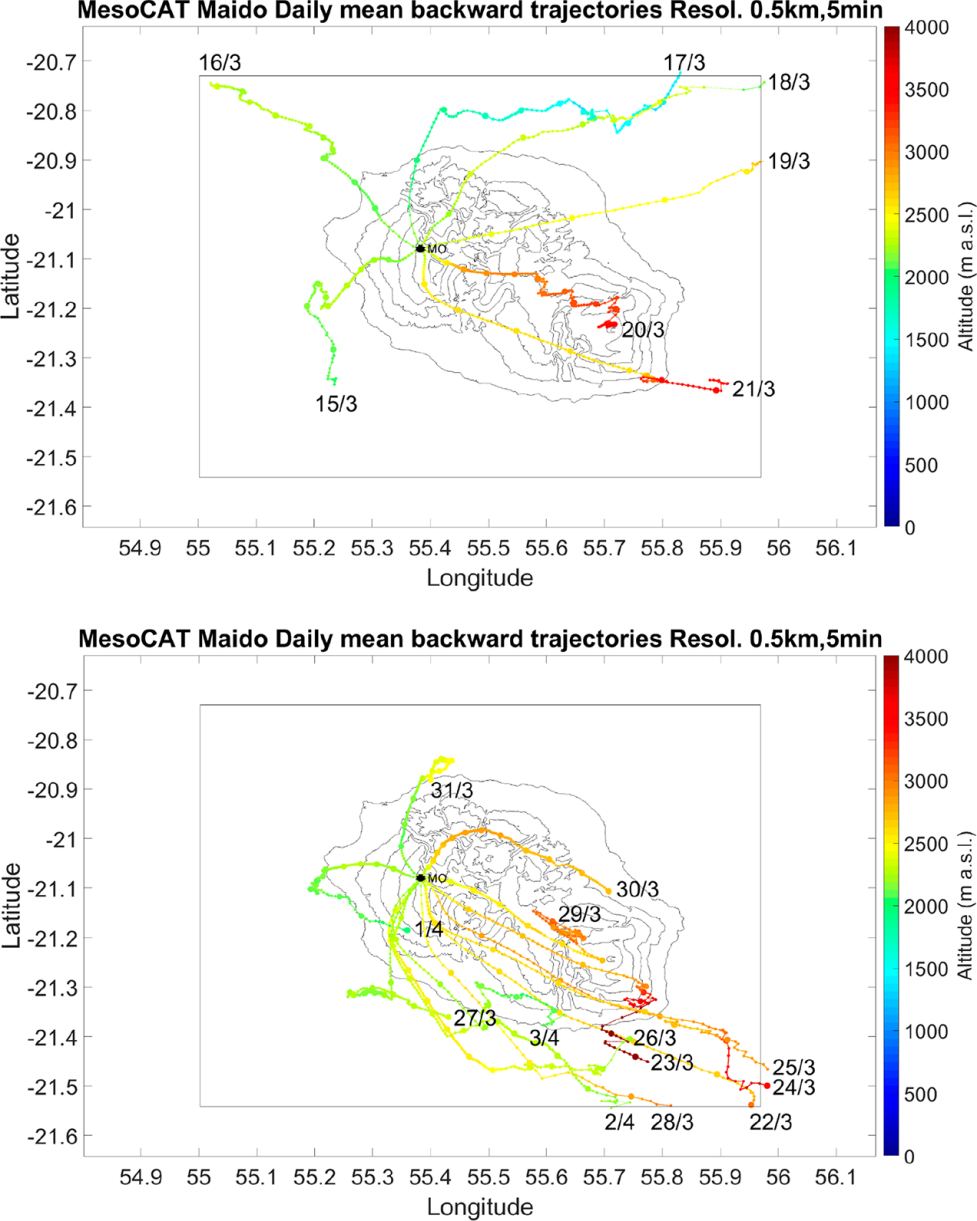
Average diurnal backward trajectories obtained from the MO for each day of our study: (top) for the days of Period 1 and (bottom) for days of Period 2 (from 3 to 16 UTC). Colors represent the altitude of air masses in m.a.s.l.

The evaluation of campaign periods also integrates the backward trajectories’ analysis results. Figure 3 shows the average daily (3 to 16 UTC, 7 to 20 LT) backward trajectories (12 h) obtained during the two periods of the campaign. During Period 1, air masses arrived from long-range distances (most of the computed points out of the model domain) and high altitude, except for the 17th of March. On the first days of Period 2, high altitude air masses were also observed, but low altitude air masses were measured at the end of the field campaign (except for the 30th of March). In the second period, the direction of air masses was more homogeneous, mainly arriving from the east–southeast of the island. More details about daily backward trajectories are presented in Figure S10a–c.

The differences in the chemical composition of NR-PM_1_ aerosols were evaluated for each of these periods and will be discussed in the following sections. In addition, relationships between the chemical species and PMF factors were investigated for the two different periods, daily averages, and daytime concentrations (9 to 19 LT, under boundary layer conditions).

### 3.3 NR-PM_1_ (ToF-ACSM) Chemical Composition

Although filter measurements provide detailed information on aerosol chemical composition over the campaign period, they lack the temporal resolution to assess the evolution of the chemical and physical processes throughout the day. During this campaign, in parallel to offline filters, the chemical composition of the NR-PM_1_ species was measured using a ToF-ACSM. The average chemical composition of the PM_1_ aerosol was dominated by SO_4_^2–^ (57.3%), followed by organics (23.3%), NH_4_^+^ (14.2%), and NO_3_^–^ (2.2%).

**Figure 4:**
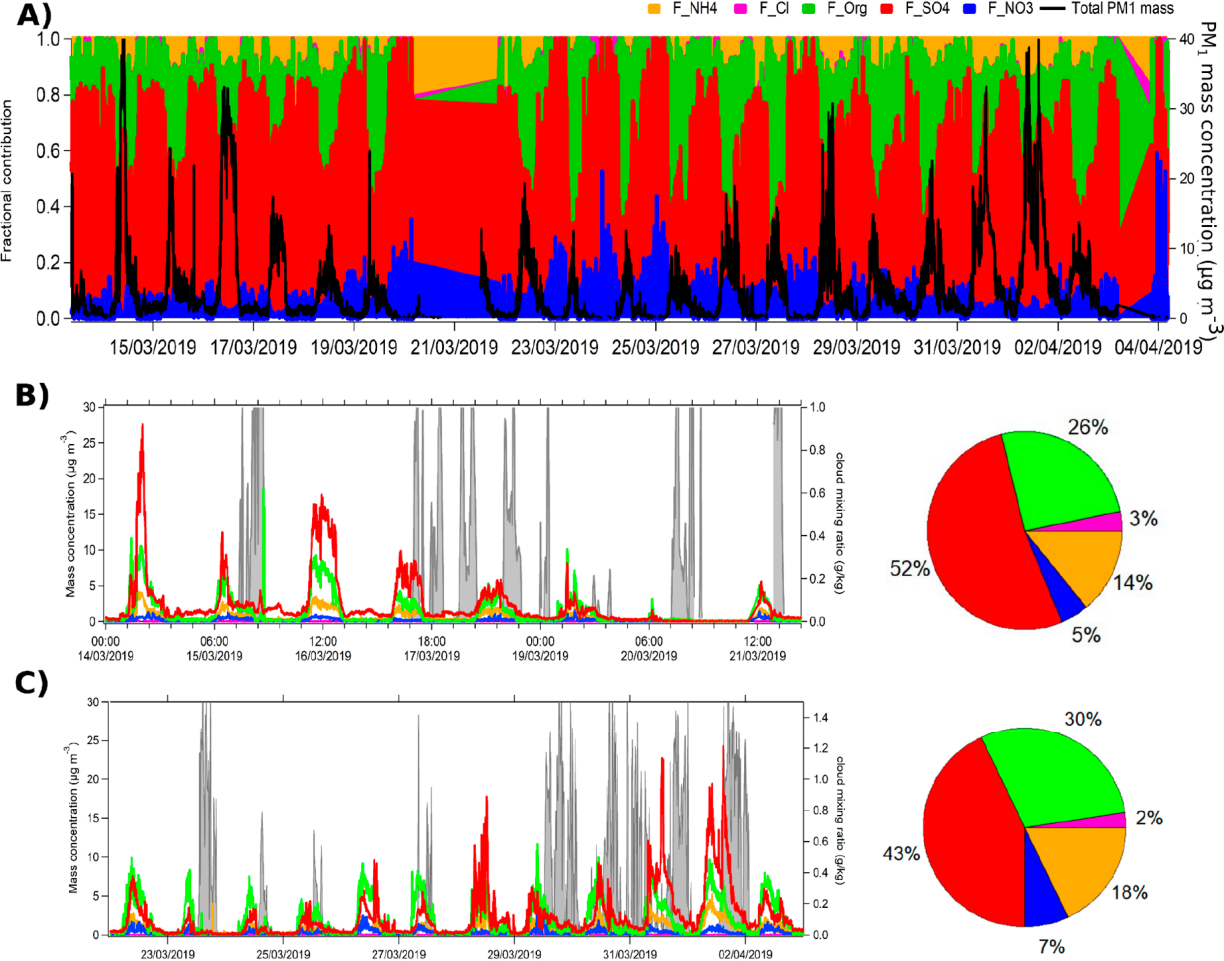
(A) Contribution of the different NR-PM_1_ species as a function of the field campaign duration; mass concentration of the NR-PM species for the (B) first and (C) second part of the field campaign. Gray shaded areas represent the cloud mixing ratio obtained from Meso-CAT backward trajectories from the MO every 15 min.

Average mass concentrations were 4.6 ± 6.2 μg m^–3^ with maximum daily concentrations always greater than 10 μg m^–3^, but nighttime concentrations were close to the detection limit of the instrument (Figures 4A and S11). The temporal variation of the different chemical species shows a strong increase in aerosol concentration at 8 a.m. LT, corresponding to the rising boundary layer. It is assumed that the site is in the free troposphere during the evening and nighttime hours, confirmed by a decrease in aerosol concentrations. A detailed description of the site dynamics is available in Lesouëf et al.^39,40^ The fractional contribution of species over time (Figure 4A) shows that the mass concentrations of organic species are negligible during the nighttime hours (Figure S12), suggesting that few OM contributions were from the FT. This is different from what was observed in the PM_10_ filters, which showed that, despite a considerable decrease in concentration, the relative contributions of all species remained constant. This might suggest that the organic material observed on the filters are particles in the supermicron mode (or that the limit of detection of the ToF-ACSM does not allow us to measure the organic species).

**Figure 5:**
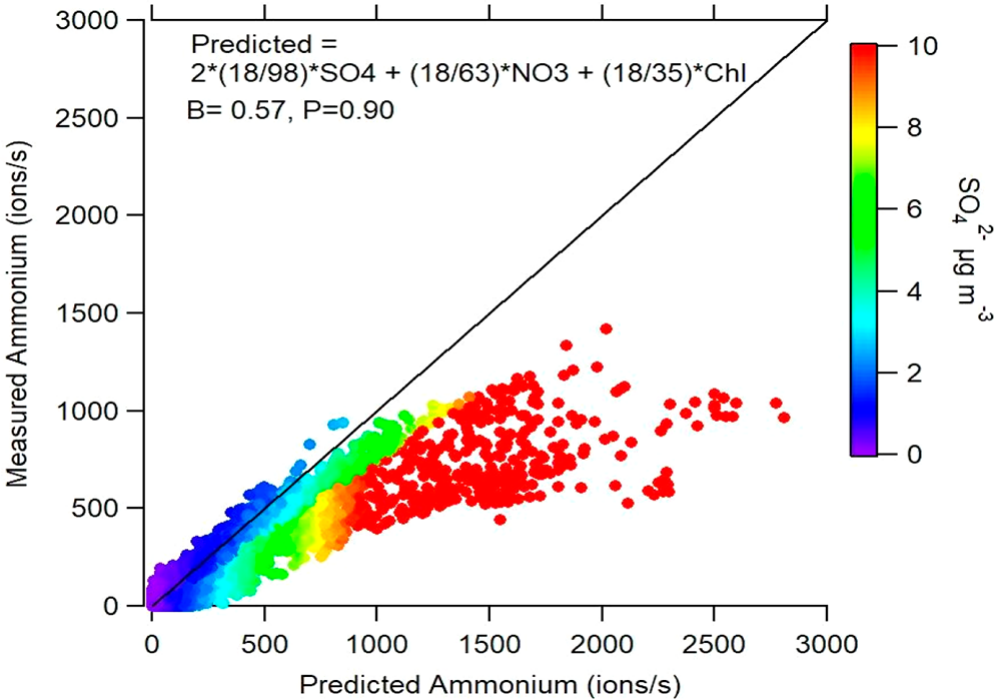
Scatter plot between measured ammonium and predicted ammonium, colored by SO_4_ mass concentrations.

Differences in the PM_1_ compositions are observed between the two sampling periods with a decrease in the sulfate content during Period 2, which is compensated by a slight increase in organics, ammonium, and nitrate (Figures 4C and S11). Nevertheless, the average contribution of the organic aerosols was considerably lower than previous NR-PM_1_ measurements in the Northern Hemisphere.^1,5,6^ These studies showed a clear dominance of organics in continental and urban sites. The dominance of sulfate aerosols is characteristic of the submicron aerosol composition collected at altitude and coastal or oceanic stations^102^ (Figure S13). The high sulfate contributions in these remote and marine locations are most likely a result of the low contribution of gas-phase precursors to form organic and nitrate aerosols. More precisely, the low NOx (and VOC) abundance leads to a decrease in HNO_3_ formation and an increase in the production of peroxides, making more OH available for the formation of SO_4_ from SO_2_.^103^ In marine environments, we might additionally expect that contributions from shipping SO_2_^104^ or marine phytoplankton emissions of dimethyl sulfide^105^ might contribute to the formation of sulfate aerosols. These later pathways are more difficult to identify at the MO station, given the low SO_2_ concentrations (0.26 ± 0.29 ppb) and low DMS concentrations measured by the PTRMS (lower than the detection limit). Recent observations^106^ highlighted the important role of one of the DMS oxidation products, hydroperoxymethyl thioformate (HPMTF-HOOCH_2_SCHO), in the formation of particulate sulfate. However, the concentrations of these species were in the ppt ranges and are below the limit of detection of the instruments used in this work. The high sulfate and low ammonium mass concentrations suggest that the aerosol sampled at the MO is acidic, containing forms of sulfate other than (NH_4_)_2_SO_4_, such as NH_4_HSO_4_, or eventually in the form of organosulfates.^46,107^ This is illustrated using the measured ammonium vs predicted ammonium ratio (if sulfate, nitrate, and chloride were only present as (NH_4_)_2_SO_4_, NH_4_NO_3_, and NH_4_Cl; Figures 5 and S14) and shows a slope of 0.57 with large deviations from the 1:1 line occurring at SO_4_^2–^ mass concentrations greater than 2 μg.

**Figure 6:**
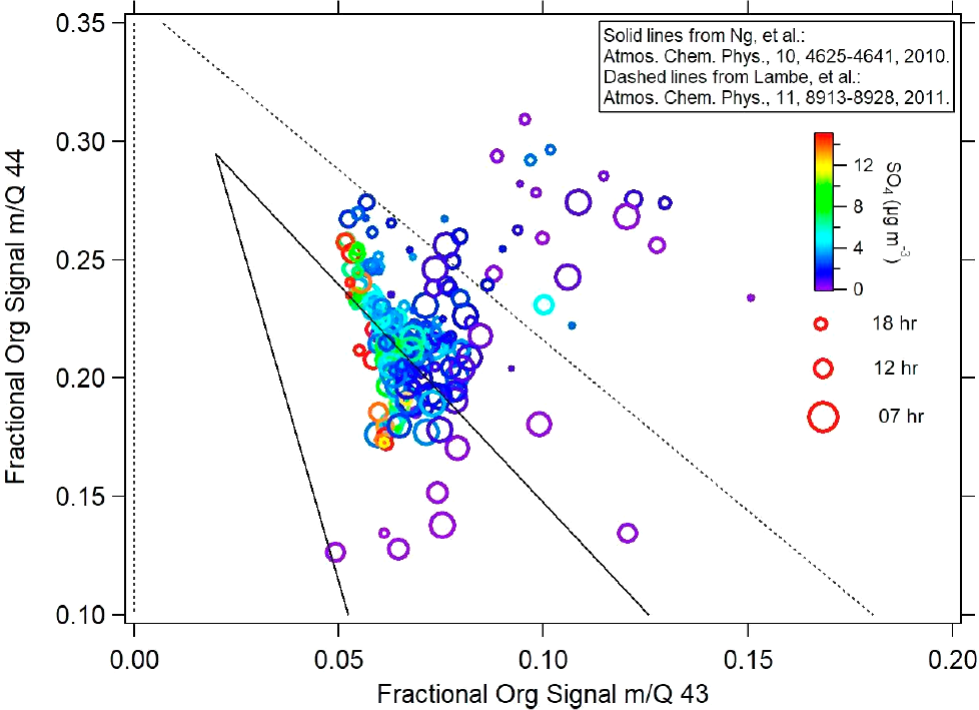
Fractional organic signal at *m*/*z* 44 vs *m*/*z* 43 (hourly averaged), color-coded by sulfate mass concentrations.

In order to extract additional information from the organic mass spectra, typical fragmentation patterns were examined. The plot of the fractions of two major mass spectral peaks at *m*/*z* 44 [CO_2_^+^] and *m*/*z* 43 [C_2_H_3_O^+^] places the organic aerosol in a triangular space in which organic aged aerosols are represented at the highest triangle point onward^108^ (Figure 6). The hourly averaged data points, including only those measurements acquired during the day (7 a.m. to 5 p.m. LT), are colored by SO_4_^2–^ mass concentration (Figure 6). The highest SO_4_^2–^ concentrations are generally associated with organic species with a range of f44 values from 0.18 to 0.27 but only a narrow range of f43 values (0.05 to 0.07). The higher f43 values occurred mainly during the early hours of the day (6 to 8 am LT), while the maximum SO_4_^2–^ values were mainly observed in the afternoon (the smallest points). In previous studies, the position of the points in the triangular space have provided information on the potential sources, whereby data points lying on the right side have been associated with a biogenic influence^5,108^ and those in the lower left to the middle side of the triangle show evidence of fresh anthropogenic emissions.^108^ Thus, our results suggest the potential influence of biogenic organics with most of the points situated on the right side of the triangle. Since photochemical aging leads to an increase in f44,^109−111^ the f44 axis can be considered an indicator of atmospheric aging. Recent studies have also reported the relationship between f44 and dicarboxylic acids due to thermal decarboxylation processing.^63,108^ We additionally observe higher f44 fractions during the latter period of the field campaigns (not shown here), which agrees with the increase of the dicarboxylic acids (such as malic acid and oxalic acid) observed in the PM_10_ composition (Figure S8).

**Figure 7:**
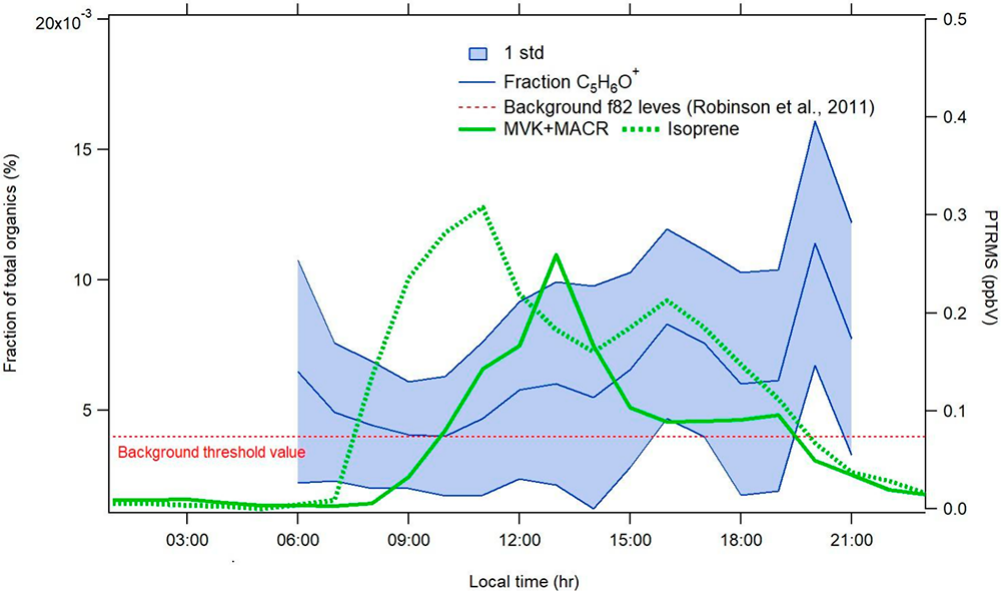
Average diurnal variability (LT) of MVK+MACR and isoprene concentrations (green lines) and the *m*/*z* 82 values relative to the total organics (blue line). Dashed red line represents the f82 background values.

When one considers the contribution of isoprene species coupled with the high aerosol acidity, high sulfate mass concentrations, and low NOx mixing ratios (<1 ppbv^112^), the atmospheric conditions encountered at the MO are suitable for the formation of isoprene-derived epoxydiol organic aerosols (IEPOXOA). These organic species can be formed from methyl vinyl ketone (MVK), methacrolein (MACR), and isoprene hydroxy hydroperoxide (ISOPOOH).^61^ Reactions of oxidized isoprene hydroxy hydroperoxide (ISOPOOH)^127^ with acidic sulfate aerosols can result in the formation of IEPOX-derived SOA.^128−130^ Although IEPOXOA gas-phase uptake is favored by acidity, aqueous phase neutral ammonium sulfate can also participate in IEPOXOA formation.^113,114^ IEPOXOA has been principally identified in pristine environments, such as the Amazon,^107,112^ but has also been identified in urban environments^61^ under low NOx conditions.^115,116^ Signature peaks for IEPOXOA in the aerosol mass spectrometer are *m*/*z* 82 (C_5_H_6_O^+^) and *m*/*z* 53 (C_4_H_5_^+^).^112^ Background values of the f(*m*/*z* 82) peak are considered to be approximately 0.4%, depending on the type of dominant OA source.^115,117^ Therefore, any value above 0.4% might suggest the presence of isoprene-derived OA (Table S2). Figure 7 presents the diurnal variability of f(*m*/*z* 82) (C_5_H_6_O^+^) relative to the total organic mass, together with isoprene and its oxidation product (MVK+MACR) concentrations. During this work, the range of f(*m*/*z* 82) values varied from <0.01% up to 1.8% with the highest values observed when isoprene and MVK+MACR concentrations were the highest (Figure 7) and when the aerosol acidity was the highest (the lowest NH_4_^+^ measured/predicted ratio), confirming the link between isoprene oxidation products and the formation of secondary biogenic OA.

### 3.4 Positive Matrix Factorization (PMF) Results

**Figure 8:**
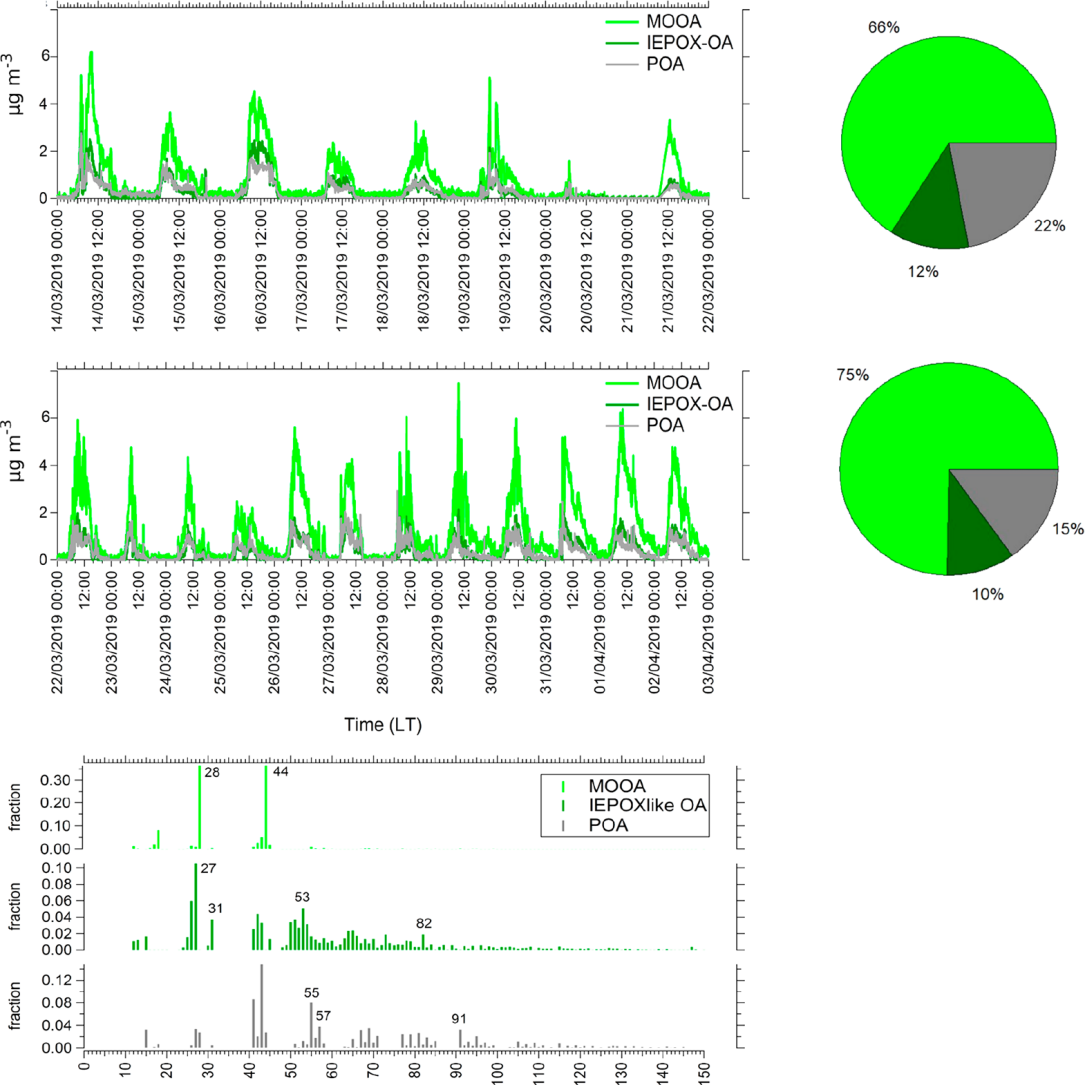
PMF factor contribution time series and relative contribution during the two campaign periods.

PMF was performed on the organic mass spectrum from *m*/*z* 10 to *m*/*z* 150; signals after *m*/*z* 150 were excluded from the analysis because of low signal-to-noise ratios. A three-factor solution (Figure 8) had a *Q*/*Q*_exp_ ratio of 0.86 and characterized up to 80% of the total organic aerosol fraction measured. Factor 1 contributed on average 70.5% to the total resolved PMF solutions, had dominant *m*/*z* fragments at *m*/*z* 28 (36.3%) and *m*/*z* 44 (36.3%), correlated well with reference mass spectra for MOOA (*R* = 0.97), oxalic acid (*R* = 0.76), malonic acid (*R* = 0.76), and alanine (*R* = 0.65), and was therefore interpreted as aged/oxidized organic aerosols. In addition, it had an estimated O/C ratio of 1.59 and H/C ratio of 1.5.^118^ Factor 2 contributed 11% on average to the total resolved PMF solutions, correlated with reference mass spectra for IEPOXOA (*R* = 0.3), and had prevalent peaks at *m*/*z* 53 (5%) and *m*/*z* 82 (1.19%) compared with the other resolved mass spectra. The low correlation is principally a result of the missing *m*/*z* 44, which was not attributed to this factor during the PMF analysis. The temporal evolution of this IEPOXOA factor and biogenic age estimation (MVK+MACR+ISOPOOH/isoprene; Figures S16 and S17) were similar (*R* = 0.41, in Period 1, Table 1), which is a good indication that this factor could be interpreted as a secondary organic aerosol derived from biogenic aerosols. However, this species did not contain any *m*/*z* 44. This may result from the PMF solution allocating incorrect amounts of *m*/*z* 44 to the different factors; similar observations have been made on other databases analyzed with this instrument.^13^

**Table 1:** Pearson Correlation Coefficients (*R*’s) Obtained for Daytime Only (7 to 17 LT) for Each Observation Period^a^

	SO_4_	NH_4_ ratio	Frac_accum	Frac_Aitken	Frac_Nucleation	MVK+MACR/isoprene	succinic acid	oxalic acid
Period 1, *n* > 1064								
POA	0.68	–0.06	0.23	–0.37	0.16	0.41	0.48	0.08
MOOA	0.79	–0.08	0.13	–0.12	0.02	0.28	0.7	0.21
IEPOXOA	0.86	–0.23	0.33	–0.33	0.07	0.41	0.44	0.05
Period 2, *n* > 1300								
POA	–0.21	0.25	–0.28	–0.37	0.43	0.003	0.83	0.83
MOOA	0.18	0.26	0.21	–0.06	–0.1	–0.13	0.83	0.68
IEPOXOA	0.18	0.2	0.09	–0.22	0.08	–0.05	0.87	0.73

a The number of data points was greater than 1000, so all correlations greater than 0.10 are significant (>0.001).

Factor 3 contributed to 18.5% on average of the total resolved PMF solutions and showed a good correlation with palmitic acid (*R* = 0.82) and oleic acid (*R* = 0.79). Furthermore, its mass spectrum features strong peaks typical of saturated hydrocarbon compounds like *m*/*z* 55 (C_4_H_7_^+^), *m*/*z* 57 (C_4_H_9_^+^), *m*/*z* 67 (cycloalkanes, C_5_H_7_^+^), *m*/*z* 69 (alkenes), and *m*/*z* 85 (saturated normal and branched alkanes, C_6_H_13_^+^) as reported by Lun et al.^119^ at Okinawa. Furthermore, we notably identify strong similarities with the primary marine aliphatic-rich organic aerosol factor (containing signals at *m*/*z* 55, *m*/*z* 57, *m*/*z* 71, and *m*/*z* 91) identified from sea spray emissions in Freney et al.^13^ Thus, this factor is identified as POA due to a low *m*/*z* 44 and high *m*/*z* 43 (14.8%) peak typical of less-oxidized organic aerosols. In addition, this factor had a calculated O/C of 0.19 within the range of values observed for primary species.^118^ On the other hand, aliphatic hydrocarbon signatures (*m*/*z* 115, *m*/*z* 117, *m*/*z* 119, and *m*/*z* 131)^59,120^ are present in low amounts (<2%). Despite this, the contribution of anthropogenic VOC is too low to consider a real primary anthropogenic source, which might suggest that biogenic aliphatic hydrocarbons other than alanine or glycine could contribute to the POA fraction.

Differences in the contribution of PMF factors were observed between the sampling periods. An increase in the MOOA factor is observed over time with a contribution of 66% during Period 1 and 75% during Period 2. The opposite trends were observed for IEPOXOA and POA factors in which contributions were 12% and 22% in Period 1 and 10% and 15% in Period 2, respectively (Figure 8). The increase of more oxidized aerosols in the second period agrees with the increase of organic acid concentrations, such as oxalic, malonic, and succinic acids, observed from offline filter analysis (Table 1). In addition, those compounds are associated with the secondary formation in aqueous or particulate phases^121^ and, together with the increase of humidity and cloud events, indicate the presence of more processed air masses.

Figure S16 shows the diurnal variability of all PMF factors studied regarding periods previously defined. The MOOA factor peaked at 2.5 μg m^–3^ (0.80 μg m^–3^ on average) around 9 a.m. LT during Period 1 and at 3.5 μg m^–3^ (1.25 μg m^–3^ on average) at the same time during Period 2 (Figure S16). The IEPOXOA factor diurnal profile did not present any substantial difference between the two campaign periods, showing similar maxima at 9 a.m. LT and average values of 0.50 and 0.53 μg m^–3^, respectively (Figure S16). The diurnal POA profile remained relatively constant with average mass concentrations of 0.34 and 0.44 μg m^–3^ during the first and second periods, respectively (Figure S16). However, its diurnal profile shows a 3 h plateau in Period 1, indicating that the POA contribution was somehow constant, which did not occur for so long during Period 2. On the other hand, a second peak is observed at noon (12 pm LT) on Period 2. Maximum values for POA were slightly higher in Period 2 at 7 a.m. LT.

The contributions from free tropospheric air masses (nighttime data) are not provided here, as the organic NR-PM1 concentrations were close to the instrument’s detection limit. We, therefore, only evaluate the correlation between PMF factors and other collocated measurements under boundary layer conditions (from 8 am to 7 pm LT). None of the factors correlated with NO_x_, CO, and SO_2_, showing the low influence of anthropogenic sources on these factors. Interestingly, correlations with sulfate in Period 1 (*R* = 0.79 for MOOA, *R* = 0.68 for POA, *R* = 0.86 for IEPOXOA) tend to show that sulfate, IEPOXOA, and MOOA could share common sources and/or formation processes, as discussed earlier (Table 1). In Period 2, these correlations present lower coefficients (*R* = 0.18 for MOOA, *R* = −0.21 for POA, *R* = 0.18 for IEPOXOA), which could be related to the changes in the sources of sulfates and organics. This pattern is also observed in the correlation of PMF factors and VOCs, which show an overall better relationship during Period 1 than Period 2. The concentration ratio of isoprene to its oxidation products (MVK/MACR/ISOPOOH) is useful to determine the processing of biogenic emissions, correlated well with the IEPOXOA and POA. Considerably better correlations are observed during P1 than in P2 (Figure S17) and with isoprene and its oxidation products separately. The IEPOXOA factor correlates well with sulfate (*R* = 0.86) and does not correlate with the NH_4_ ratio (*R* = −0.23) in Period 1, suggesting a local production of IEPOXOA, whereas the weak correlation of IEPOXOA with sulfate (*R* = 0.18) observed in Period 2 suggests that the IEPOXOA was either transported from other source regions or chemically aged. This hypothesis is also supported by the good correlations with succinic (*R* = 0.87) and oxalic (*R* = 0.72) acids observed during Period 2 (compared with Period 1). Both species were tracers for aged air masses. Our results indicate the presence of different secondary formation processes in both periods of the field campaign.

Despite the much lower VOC concentrations than those usually observed in anthropogenic environments, slightly better correlations between POA and primary VOC are observed (such as benzene, *R* = 0.83), suggesting the presence of less processed emissions. The strong negative correlation between secondary organic aerosol (notably IEPOXOA, *R* = −0.47) and the ratio of measured to predicted NH_4_ (NH_4_ ratio, Table 1) were obtained in Period 1. These correlations were positive but weaker during Period 2 (IEPOX, *R* = 0.20) (Table 1). This confirms previous observations showing that the formation of IEPOXOA favors acidic conditions.

### 3.5 Aerosol Processing under Cloud Conditions

Case study periods were selected to evaluate the changes in aerosol chemistry due to cloud processing. The modeled Meso-NH cloud mixing ratio (g kg^–1^) was used to identify the periods when air masses were affected by cloud processing. These results show the systematic presence of cloud and fog events in the air masses arriving at the MO after the 22nd of March, except for the 1st of April. From the 22nd to 28th of March (Period 2a), cloud episodes mainly occurred for air masses arriving during evening/nighttime hours when aerosol mass concentrations were the lowest; however, from the 29th onward (Period 2b), several of these episodes occurred during the day.

**Figure 9:**
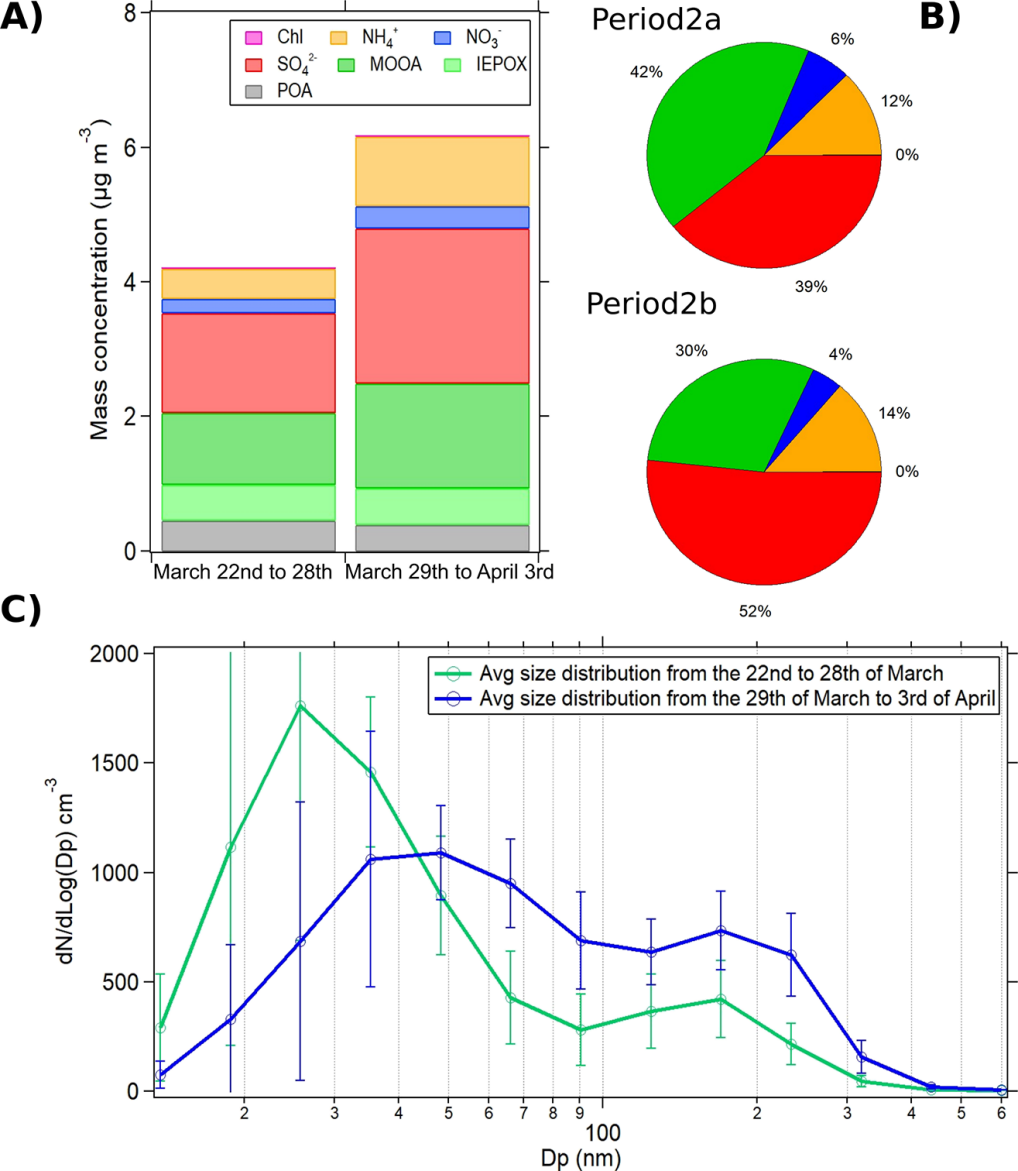
(A) Concentrations of NR-PM_1_ chemical species and PMF factors, (B) relative chemical contribution of NR-PM_1_ aerosols, and (C) average size distribution of aerosols during Period 2a (March 22 to 29) and Period 2b (March 29 to April 3).

**Figure 10:**
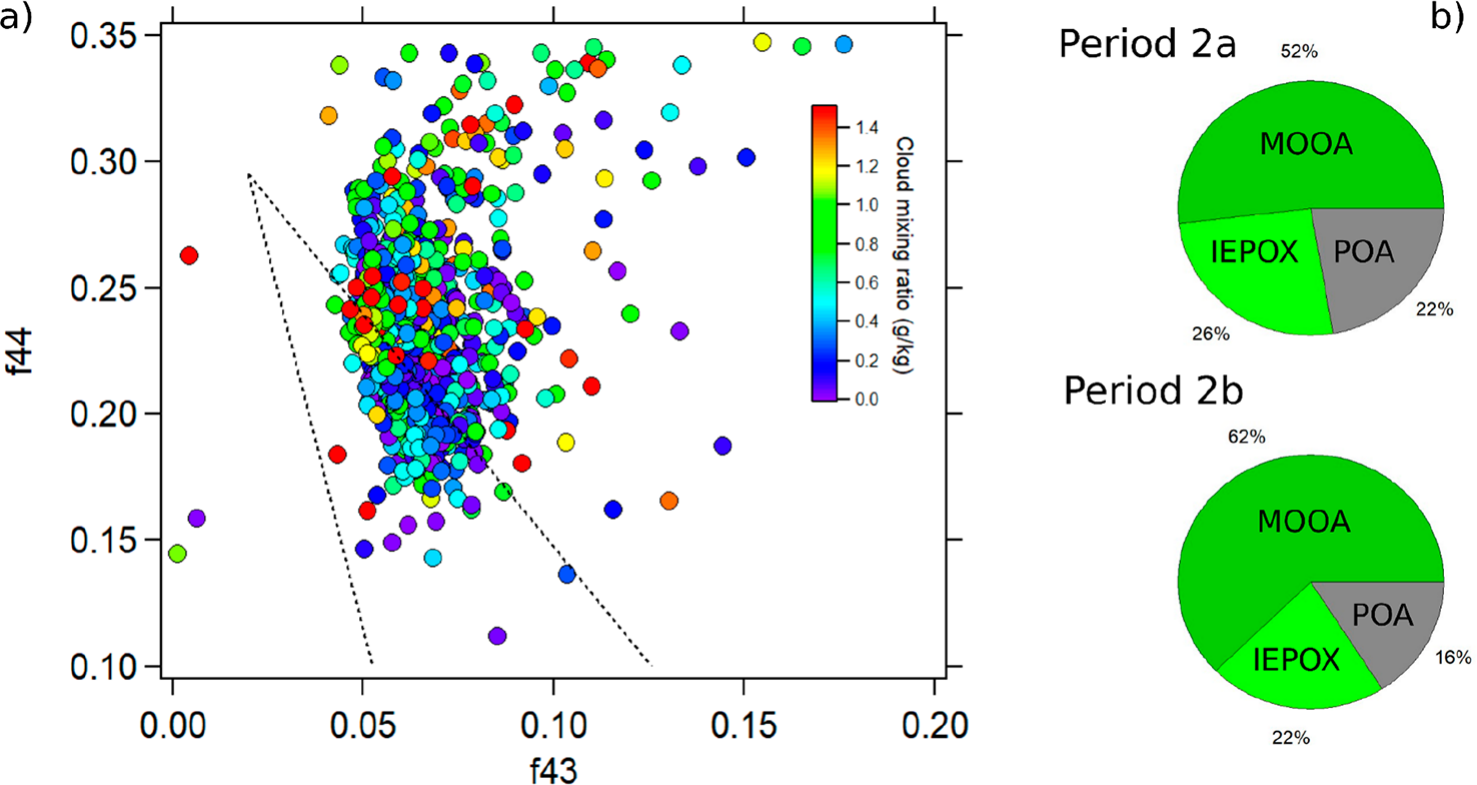
(a) Relationship between f44 and f43 color-coded by the estimated cloud mixing ratio obtained from the Meso-CAT model. (b) Contribution of PMF factors during Period 2a and Period 2b.

From the 29th of March onward, a 10% increase in the contribution of SO_4_^2–^ PM_1_ aerosols is observed (Figure 9A,B), which due to the higher presence of clouds in the air masses, could indicate an aerosol aqueous phase processing.^21^Figure 10a shows the relationship between f44, f43, and the estimated cloud mixing ratio from backward trajectories. In addition, we observed that periods when cloud mixing ratios were high were also associated with the highest f44 values, suggesting increased oxidation of OA, which is confirmed by a 10% increase in the contribution of MOOA (Figures 9A and 10). Even though other photochemical processes could affect the formation of oxygenated OA, the higher presence of cloud events in the last part of the campaign suggests that cloud processing at Réunion has a potential role in forming highly oxidized organic aerosols. These observations, although low, are in line with increases reported in previous studies, where an increase (15% to 25%^27^) of MOOA aerosol was observed as a function of relative humidity and aerosol liquid water content.^20,25,26,122^

Similar changes were observed from the offline PM_10_ filter measurements with a 48% increase in sulfate and a 70% increase in ammonium concentrations, indicating that aqueous processing potentially influences all the aerosol size fractions. As previously described, oxalic acid concentrations measured in PM_10_ show a 2-fold increase during Period 2 compared to Period 1 and the concentrations of malonic and succinic acids (Figure S8). Our results indicate that in-cloud oxidation of gaseous precursors, the degradation of primary biogenic organic matter, and the condensation of soluble species onto the surface of particles (aerosol water) could have a role as precursors of oxalic acid^21,123^ that have an impact in the aerosol formation/processing at Réunion. In agreement with this hypothesis, a shift in the aerosol size distribution is also observed during this period (Figure 9C). This shift could be a result of a lower number of nucleation events (<3%) compared to that in the first period (Figure S18). However, it may also suggest that aqueous-phase processing plays a role, shifting aerosol size distributions to larger diameters with a combined increase of 15% in the contribution of Aitken and accumulation mode aerosols. This is in line with previous studies demonstrating that aqueous chemistry could lead to faster particle growth rates than photochemical activities.^124^

## 4 Conclusions

This study aimed to understand the sources and evolution of different aerosol species at a high-altitude tropical site in the Southern Hemisphere. A combination of collocated measurements was used to accomplish this work, including online and offline aerosol chemical properties, online aerosol physical measurements, and online VOC measurements of gas-phase precursors. The valuable synergy between these measurements, together with backward trajectories’ analysis, allowed for a representative chemical and physical characterization of aerosol particles to evaluate long-range transport, local sources, and the cloud-processing impacts on chemical aerosol properties.

During the whole sampling period, little or no anthropogenic or volcanic emission contributions were identified, and we conclude that the primary sources of the NR-PM_1_ aerosols were long-range transported aerosols and local natural sources. Like other remote mountain sites exposed to marine air masses, the NR-PM_1_ aerosols were dominated by sulfate species. The organic fraction of PM_1_ was dominated by the typical more oxidized organic aerosol (MOOA), followed by organic aerosol containing signature mass spectra for primary organics. Given the low contribution of anthropogenic gas-phase species and air mass history, we estimate that these primary organic aerosols originate from natural biogenic sources. These species showed strong diurnal variations with maximum concentrations during the day and minimum concentrations at night. Sulfate particles always dominated the PM_1_ concentration, but more so at night, when few or no organic aerosols were detected. Similar diurnal variations were observed on the filter measurements, but the relative contributions of all species remained the same. These results may suggest that the NR-PM_1_ organic aerosol at the MO observatory is strongly influenced by local emissions arriving at the site with the developing boundary layer.

PMF results showed that MOOA was the dominant fraction of PM_1_, increasing at the end of the sampling campaign. In addition, IEPOXOA and MOOA factors correlated well with SO_4_ in the first campaign period (Period 1). However, these correlations decreased in Period 2, suggesting that additional organic sources or atmospheric processes impacted MO. This was also confirmed by a thorough analysis of the organic composition on the PM_10_ filters, which showed a change in organic acid content with an increase of oxidation products, such as oxalic acid, by the end of the campaign. Conversely, PM_10_ aerosols presented a predominance of organics aerosols, indicating the influence of other sources in the coarse fraction.

We demonstrated the importance of incorporating air mass history into the analysis, not only in terms of air mass origin but also in terms of exposure to cloud processing. Using this data, we were able to investigate how a previous exposure to cloud events could impact the aerosol chemical and physical properties. During Period 2, we compared two subsections of the data, one that was seldom exposed to cloud processing and a second that was exposed to varying degrees of cloud processing. Moderate changes in aerosol physical and chemical properties were observed. The average size distribution changes to larger diameters during the cloud processing period (increase by 15% of Aitken and accumulation mode aerosols), and this was accompanied by changes in the chemical composition of the aerosol with increases in the contribution of SO_4_ aerosol particles and more oxidized organic aerosol. Even though there are limitations, our results assess for the first time a comprehensive data set of gases, aerosols, and cloud mixing ratios at a high-altitude tropical site in the Southern Hemisphere. This comprehensive data set, and future related analysis, can contribute substantially to the development and constraint of chemical models, allowing for a better understanding of SOA formation pathways and a better representation of multiphase processes.

Finally, this work shows the effective benefits of combining offline and online measurements. The combination of methods used in this work could be applied to other equipped observatory sites (within the ACTRIS framework, for example) to evaluate the role of cloud processing on the formation of secondary aerosol particles.

## Supporting Information Available

Instrument details, diurnal profiles of meteorological data, ACSM, VOCs, and PMF factors, composition-dependent collection efficiency (CDCE) adjustment, aerosol distribution time series, by period and comparison between DMPS and ACSM, PMF solutions, PM_10_ filter composition analysis in a diurnal and period basis, Meso-NH model and diurnal backward trajectories, relative diurnal contribution of NR-PM1 and comparison with other studies, aerosol acidity, and correlations between the iEPOX factor and biogenic age (PDF)
