# Supplementary material for: Evaluation of the Sources, Precursors, and Processing of Aerosols at a High-Altitude Tropical Site
Source: ACS Earth Space Chem. 2022 Sep 12;6(10):2412–31. doi: 10.1021/acsearthspacechem.2c00149 (PMC9590422; doi:10.1021/acsearthspacechem.2c00149)
Supplement: Supplementary file 1 — sp2c00149_si_001.pdf [file sp2c00149_si_001.pdf]

Supplementary information of

Evaluation of sources, precursors, and processing of aerosols at a high-altitude tropical site

Pamela A. Dominutti[1,2], Emmanuel Chevassus[1+], Jean-Luc Baray[1], Jean-Luc Jaffrezo[2], Agnès Borbon[1], Aurèlie Colomb[1], Laurent Deguillaume[1], Samira El Gdachi[3,4], Stephan Houdier[2], Maud Leriche[1,5], Jean-Marc Metzger[4], Manon Rocco[1,4], Pierre Tulet[3], Karine Sellegri[1], and Evelyn Freney[1*].*

[1]Université Clermont-Auvergne, CNRS, UMR 6016, Laboratoire de Météorologie Physique (LaMP), Clermont -Ferrand, 63000, France.

[2]Université Grenoble Alpes, UMR 5001, CNRS, IRD, Grenoble, 38000, France.

[3]Laboratoire d'Aérologie (LAERO), UMR 5560, Toulouse, 31400, France.

[4]Laboratoire de l'Atmosphère et des Cyclones (LACy), UMR 8105, Université de la Réunion, Saint-Denis de La Réunion, 97744, France.

[5]Centre pour l'étude et la simulation du climat à l'échelle régionale, Département des sciences de la terre et de l'atmosphère (ESCER), Université du Québec à Montréal, Montréal, H2X 3Y7, Canada.

[+] Now at School of Physics and Centre for Climate & Air Pollution Studies, Ryan Institute, National University of Ireland Galway, University Road, Galway, Ireland.

Table S1. Instrument and measurement details performed at MO during the BIO-MAIDO campaign

Instrument	Target species	Time resolution	Measurement technique
ToF-ACSM (Aerodyne)	NR-PM_1	10 minutes	Time of Flight aerosol mass spectrometer
Hi-VOL	PM_{10} composition (ions, OC, EC, organic acids, sugars)	10-12 hours	High volume impact sampler
SMPS, TSI 3010	Aerosol size distributions	8 minutes	Differential mobility particle sizer
PTR-MS	VOCs	2.7 minutes	Proton-transfer-reaction mass spectrometry
TEI49i, Thermo Fisher Scientific	Ozone (O_3)	1 minute	UV photometric
T42I, Thermo Fisher Scientific	Nitrogen oxides (NO, NO_2, NOx)	1 minute	Blue light converter (BLC) technology
T43i, Thermo Fisher Scientific	sulfur dioxide (SO_2)	1 minute	Fluorescence
Picarro G2401	methane (CH_4) and carbon monoxide (CO)	1 minute	cavity ring-down spectroscopy (CRDS) technology
Vaisala HMP45 automatic weather station	wind speed, wind direction, relative humidity, temperature, and solar radiation	1 minute	

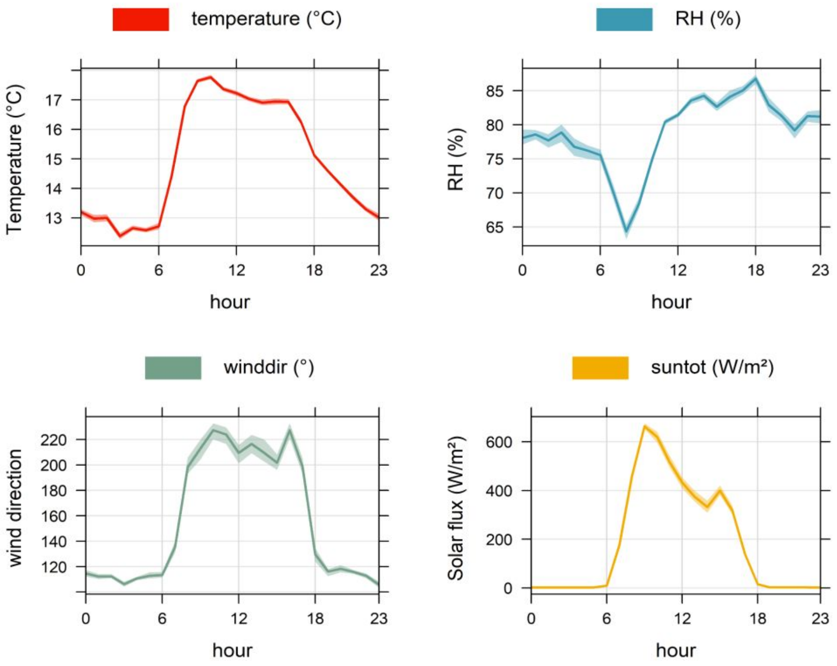

Figure S1. Average diurnal profiles (LT) of meteorological parameters (temperature, relative humidity (RH), solar flux, and wind speed). The bold lines represent the average hourly values and the surrounding shaded areas the confident intervals at 95% during the whole campaign.

<u>**Section S 1. Composition-dependent collection efficiency (CDCE) adjustment.**</u>

Below is an example of the CDCE used for this data set; during high ammonium sulfate mass fractions (ASMF) periods, a CE of > 0.45 was applied to the data. When ASMF (and Ammonium nitrate MF (ANMF) contributed <0.5 to the total PM1, a CE of 0.45 was applied to all species.

ASMF = $(SO_4+NH_4)/(Org+NO_3+NH_4+SO_4+Chl)$. CDCE = 0.45+0.5/0.6*(ASMF-0.4)

The NH_4/NH_{4pred} ratio (an indirect proxy for particles acidity, which represents the theoretical ammonium concentration needed to neutralize the inorganic species concentrations, Zhang et al., 2007) is used to verify CDCE pertinence:

$$NH_{4predicted}=2*(18/98) * SO_4+(18/63) * NO_3+(18/35) * Chl$$

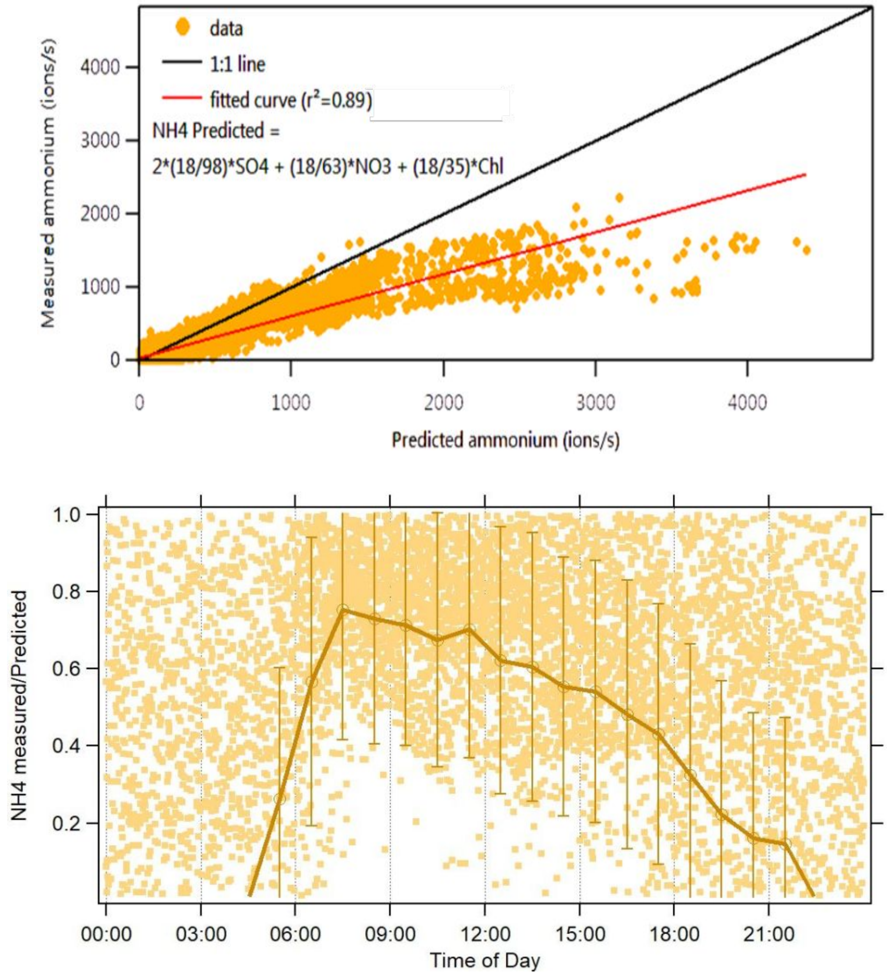

Figure S2. Scatterplot of measured and predicted ammonium (NH_4^+, yellow dots) and average diurnal profile of NH_4^+measured/predicted ratio.

Figure S2 shows that most inorganic species were neutralized as ammonium sulfate $(NH_4)_2SO_4$, ammonium nitrate $(NH_4)NO_3$Or ammonium chloride $(NH_4)Cl$. However, the 0.57 slope shows that at higher concentrations, there is a deviation away from the slope of 1, which means that there is more SO_4 or NO_3 than

needed to neutralize NH_4, from which we can suppose that SO_4 must be present in other forms than ammonium sulfate, such as H_2SO_4.

<u>Section S2. Comparison between DMPS and ToF-ACSM</u>

DMPS aerosol size distribution measurements are converted to mass concentrations and compared with ToF-ACSM mass concentrations as follows; $mass\ concentrations = \sum_{1}^{i} \frac{\pi}{6} * D_p^3 * N_i * \rho * 10^9$. Where i is a given measurement, D_p its diameter, N_i is the number of the particles (cm^{-3}), and ρ the particle's density (1.5 $g.cm^{-3}$).

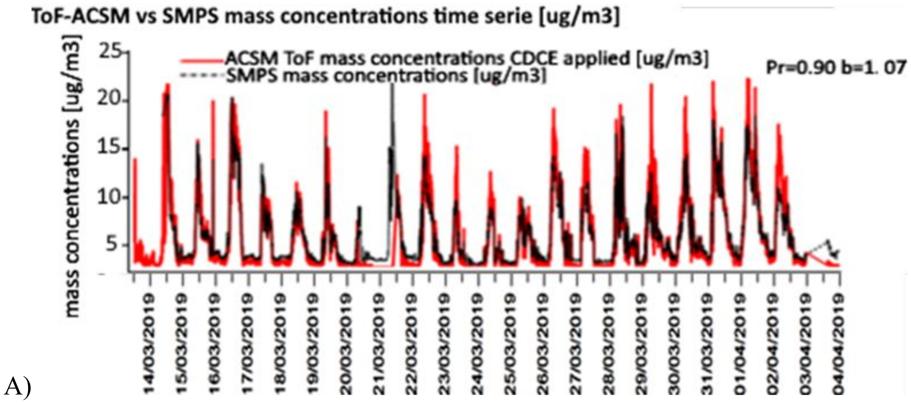

A)

Figure S3. A) Comparison of total particle mass concentration between ToF-ACSM and DMPS time series.

Aerosols' size distribution

Daily particle number concentration varied from 2000 cm^{-3} to 6000 cm^{-3}, whereas nighttime concentrations were always less than 400 cm^{-3}. Concentrations increased during morning early hours ($R = -0.45$, $p = <0.001$), which illustrates the influence of atmospheric dynamics and the rising of the boundary layer, bringing other atmospheric layers. Aitken mode aerosols (25 nm to 90 nm) contributed on average 57 ± 14 % to the total aerosol size distribution, accumulation mode aerosols (90 – 600 nm) contributed on average up to 21 ± 9%, while the nucleation mode (13.7-25 nm) contributed to 21 ± 16% (Figure S4). Maximum nucleation contributions were mostly prevalent during the early morning hours (Figure S4b), resulting from the daily new particle formation events triggered by convective uplifting of boundary layer air masses to the MO site 38,82. From 28th March onwards, we observe an increase (up to 25% ± 9.7%) in the contribution of accumulation mode aerosols. This corresponded to periods when air masses traveled from lower altitudes, passing over the forested areas and being more influenced by cloud events than during the first part of the field campaign. More discussion on the aerosol size distributions can be found in section 3.5.

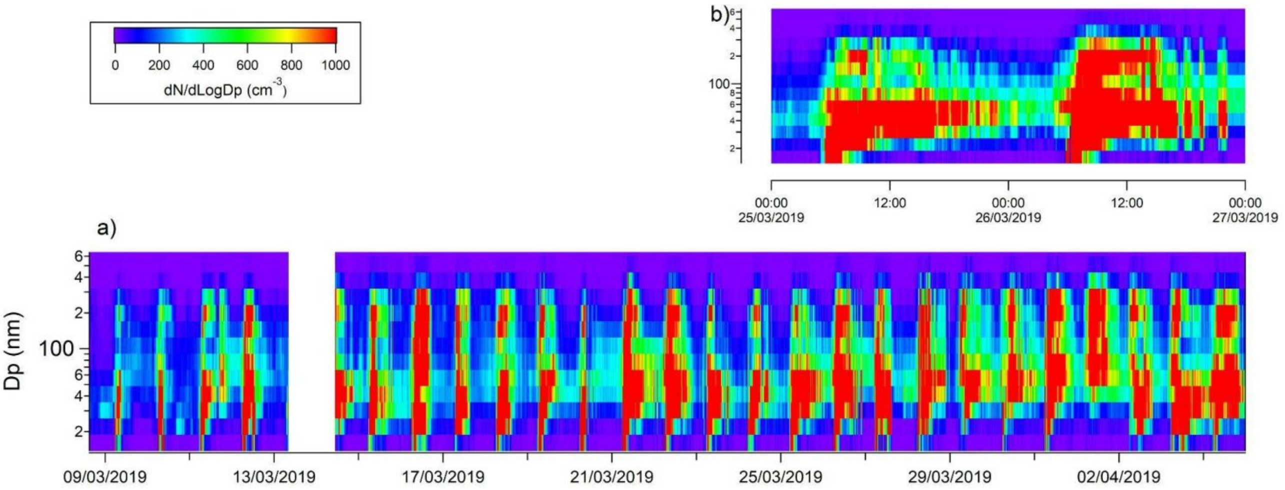

Figure S4. a) DMPS time series observed during the field campaign at MO, b) inset showing a zoom of the m diurnal variability from the 25th and 27th of March.

Section S3. Positive Matrix Factorization solutions time series and mass spectra

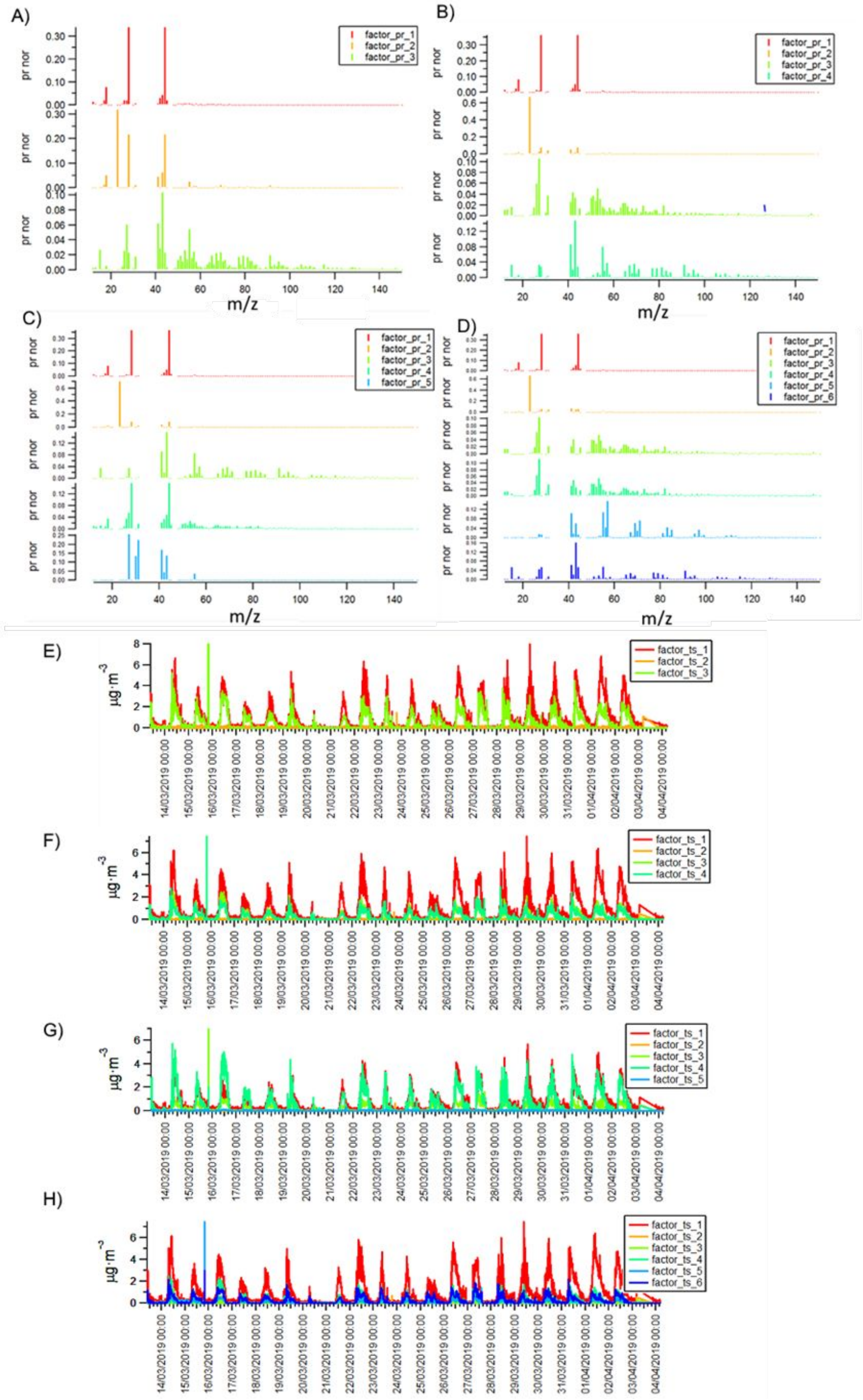

Figure S5. PMF solutions for 2 to 6 factors. A-D represent the mass spectral of each solution and E-H the factors timeseries for each PMF solution

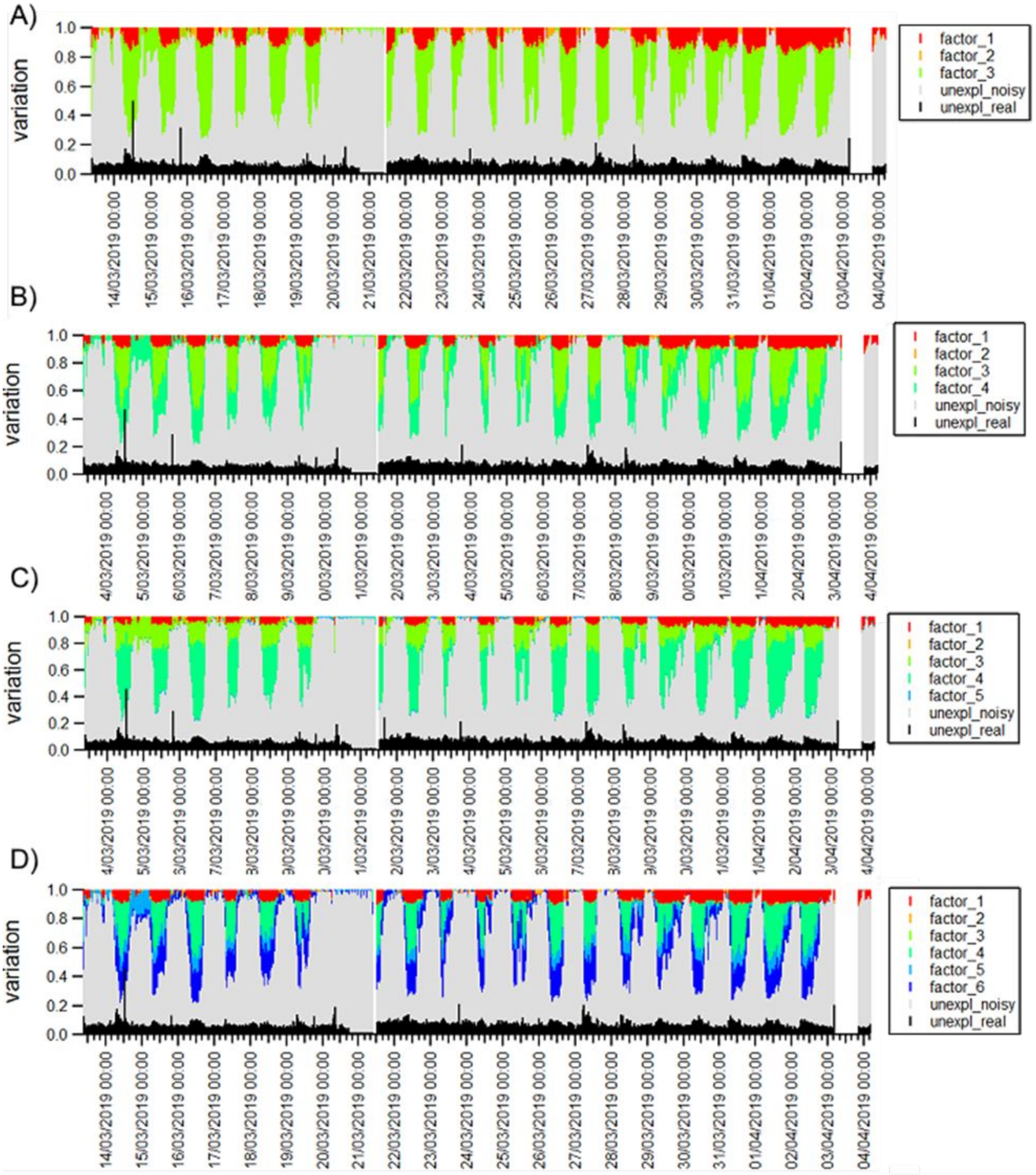

Figure S6. Relative contribution of PMF solutions (2-6 factors) including background noise

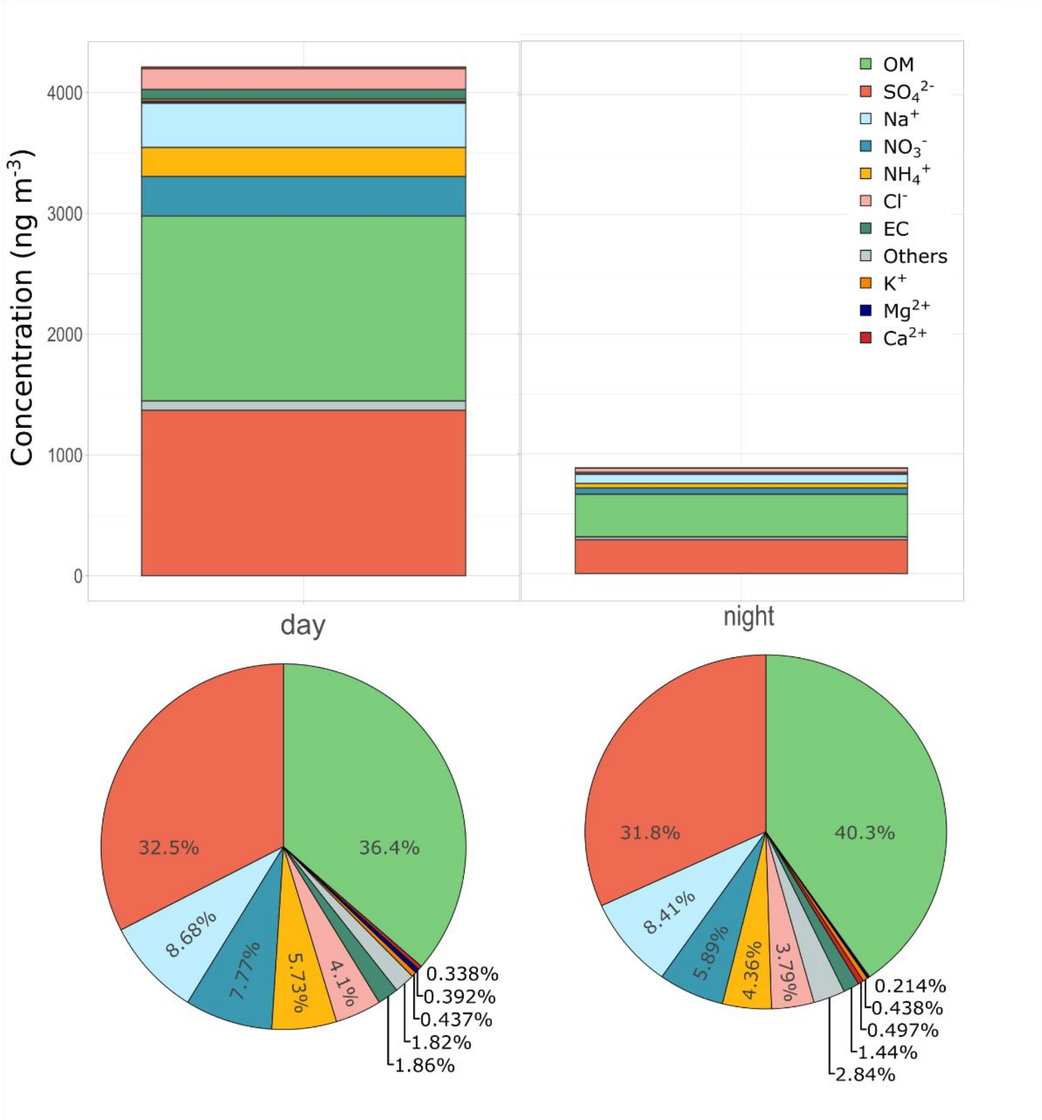

Figure S7. Day and night average mass concentrations of PM10 and day – night relative contribution for the whole BIO-MAÏDO campaign.

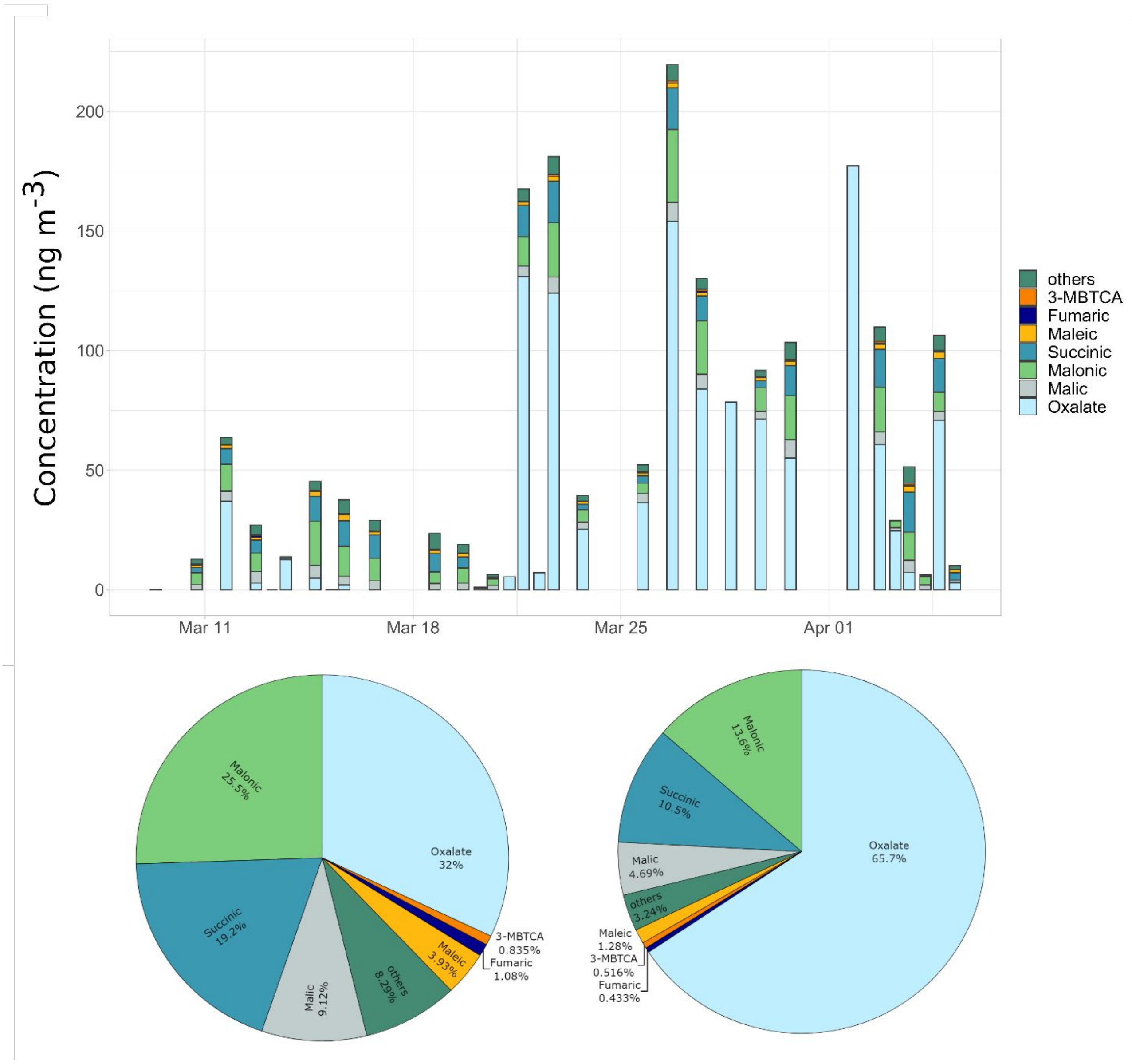

Figure S8. Organic acids time series concentrations and relative contribution for each period of measurements at MO.

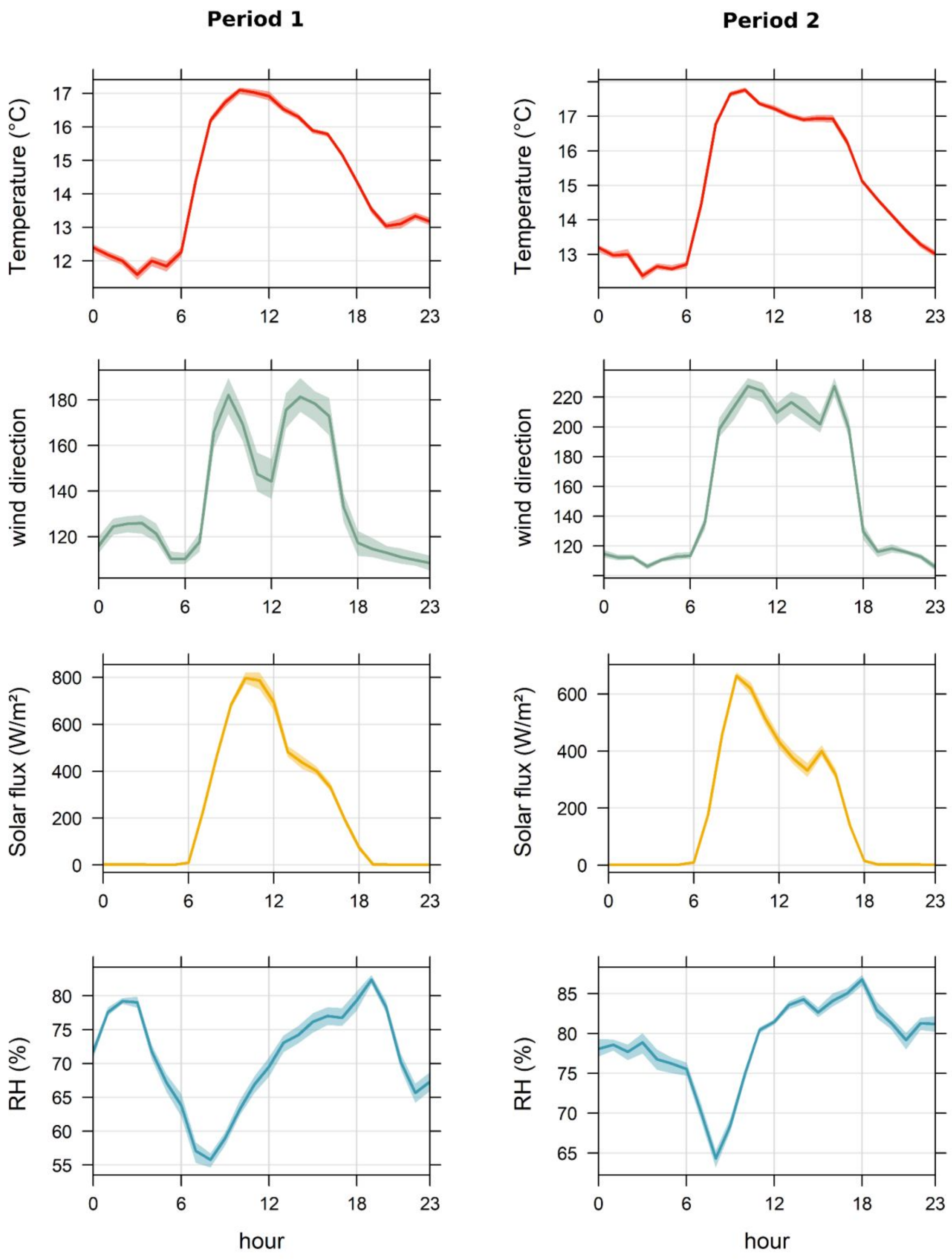

Figure S9. Average diurnal profiles (LT) of meteorological parameters (temperature, relative humidity (RH), solar flux, and wind speed), observed during the two periods of the campaign: period 1 from 8th March to 21st March (left) and period 2 from 22nd March to 4th April (right). The bold lines represent the average hourly values and the surrounding shaded areas the confident intervals at 95%.

Section S4. Meso-NH model and backward trajectories

Meso-NH simulates small scale (Large-Eddy Simulations (LES) type, horizontal resolution from a few meters) up to synoptic-scale atmospheric parameters (horizontal resolution of several tens of kilometers) and can be run in a two-way nested mode involving several nesting stages. Surface fluxes and cloud microphysics are computed using the surface model SURFEX[13] combined with the ICE-3 module[14].

The Meso-NH model has already been used to characterize air masses above Reunion Island at 500 m resolution during the FARCE campaign in 2015 and in the present work, the Meso-NH configuration used in the present work includes three nested domains at the horizontal resolutions 100, 500, and 2000 m.

All three domains include 72 vertical points, with a vertical stretched resolution decreasing with height from a few meters (near the ground level) up to 1 km (from 8 km above sea level to the top of the domain). The model results show an excellent agreement between the cloud formation on the slope between the Indian Ocean and the Maïdo observatory reproduced by Meso-Nh with the coincident observation by tethered balloon.

A high-resolution back trajectory study of this campaign focused on Volatile Organic Compounds observations has already been published in JGR-Atmosphere[15].

A)

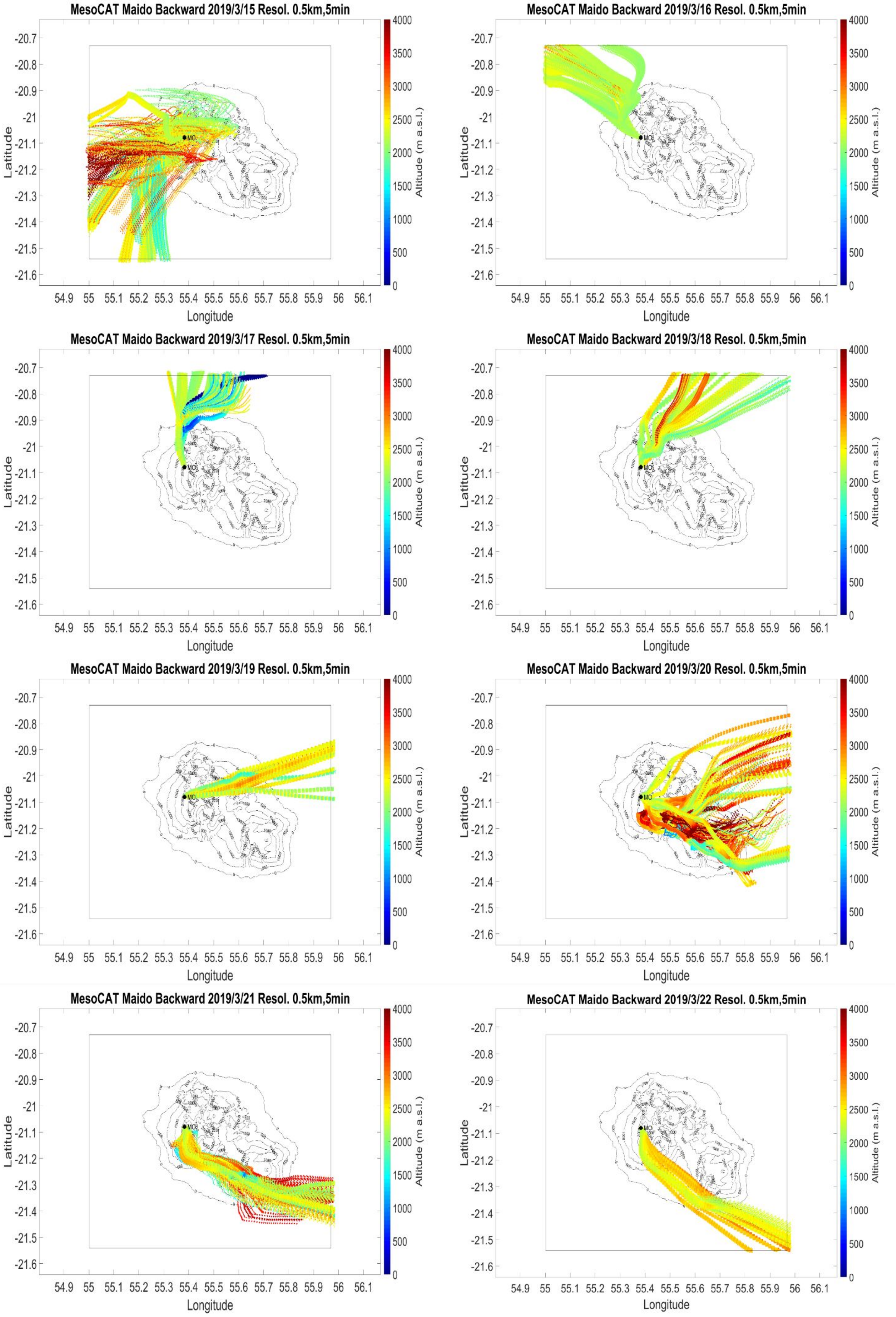
MesoCAT Maido Backward 2019/3/15 Resol. 0.5km,5min
MesoCAT Maido Backward 2019/3/16 Resol. 0.5km,5min
MesoCAT Maido Backward 2019/3/17 Resol. 0.5km,5min
MesoCAT Maido Backward 2019/3/18 Resol. 0.5km,5min
MesoCAT Maido Backward 2019/3/19 Resol. 0.5km,5min
MesoCAT Maido Backward 2019/3/20 Resol. 0.5km,5min
MesoCAT Maido Backward 2019/3/21 Resol. 0.5km,5min
MesoCAT Maido Backward 2019/3/22 Resol. 0.5km,5min
Latitude
Longitude
Altitude (m a.s.l.)
MO

B)

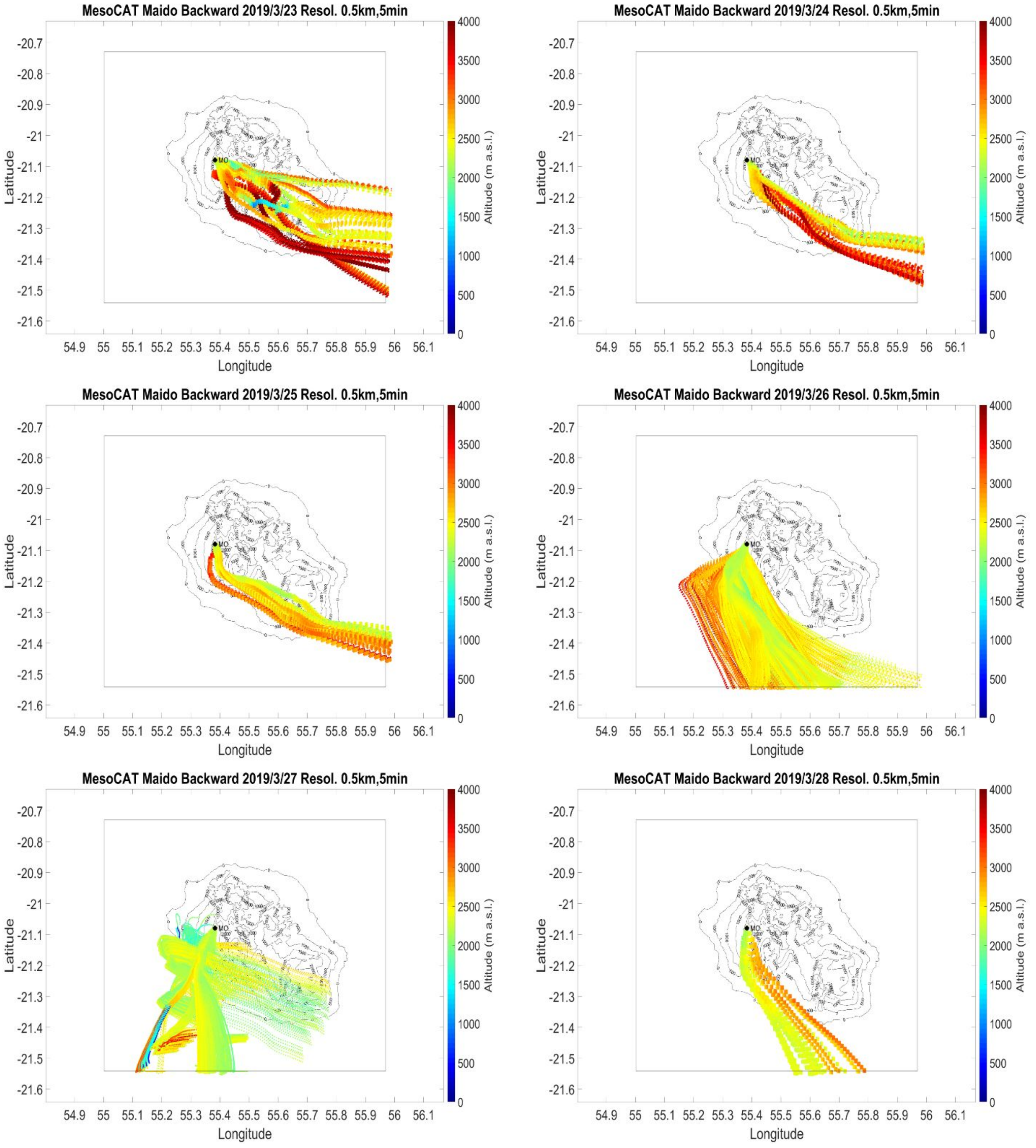
MesoCAT Maido Backward 2019/3/23 Resol. 0.5km,5min
MesoCAT Maido Backward 2019/3/24 Resol. 0.5km,5min
MesoCAT Maido Backward 2019/3/25 Resol. 0.5km,5min
MesoCAT Maido Backward 2019/3/26 Resol. 0.5km,5min
MesoCAT Maido Backward 2019/3/27 Resol. 0.5km,5min
MesoCAT Maido Backward 2019/3/28 Resol. 0.5km,5min
Latitude
Longitude
Altitude (m a.s.l.)
MO
-20.7
-20.8
-20.9
-21
-21.1
-21.2
-21.3
-21.4
-21.5
-21.6
54.9 55 55.1 55.2 55.3 55.4 55.5 55.6 55.7 55.8 55.9 56 56.1
0
500
1000
1500
2000
2500
3000
3500
4000

C)

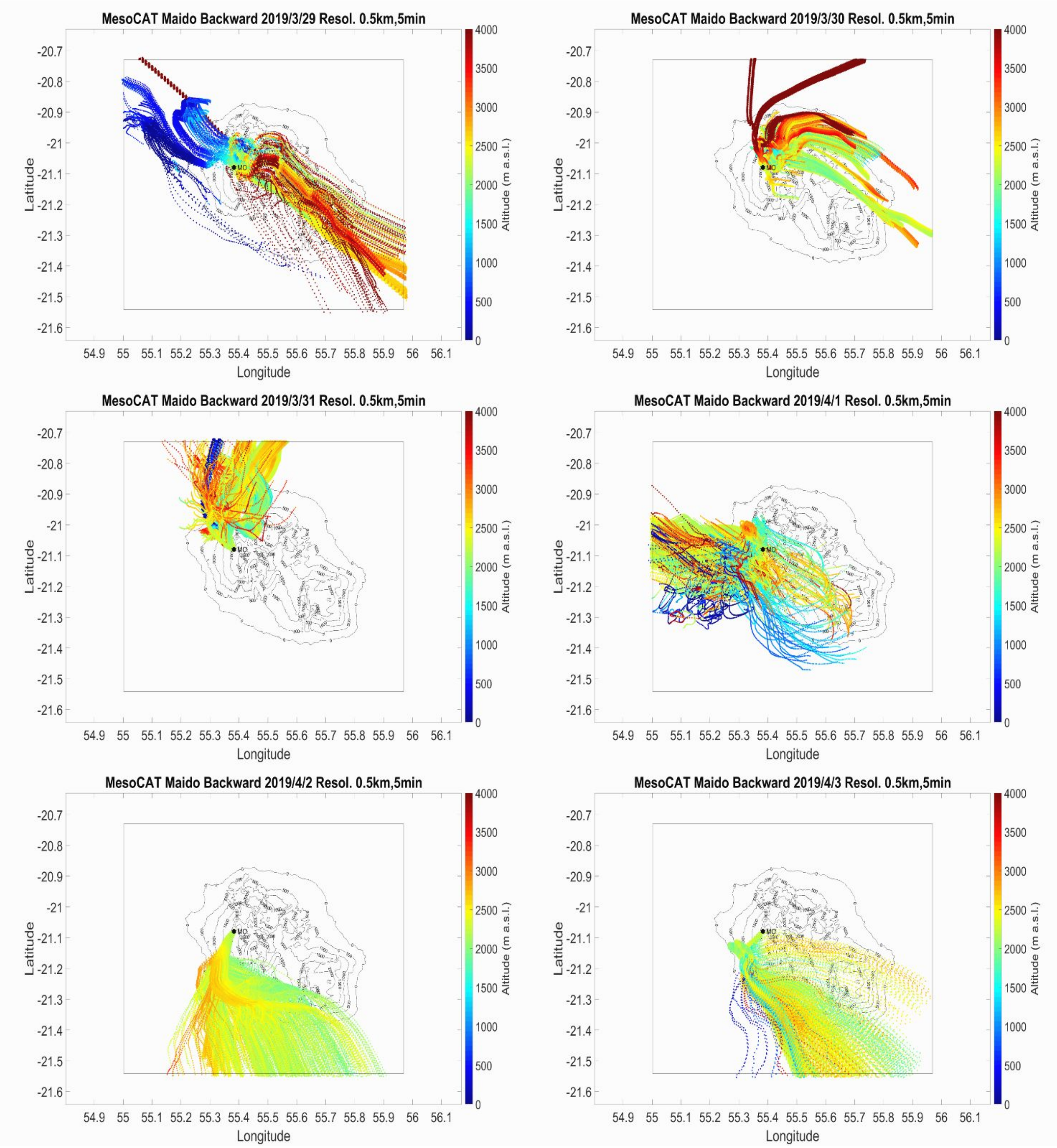

Figure S10. Diurnal backward trajectories obtained from MO during different period of the field campaign, A) Period1 (15th to 22nd Mach), B) Period2a (23rd to 28th March) and C) Period 2b (29th March to 3rd April).

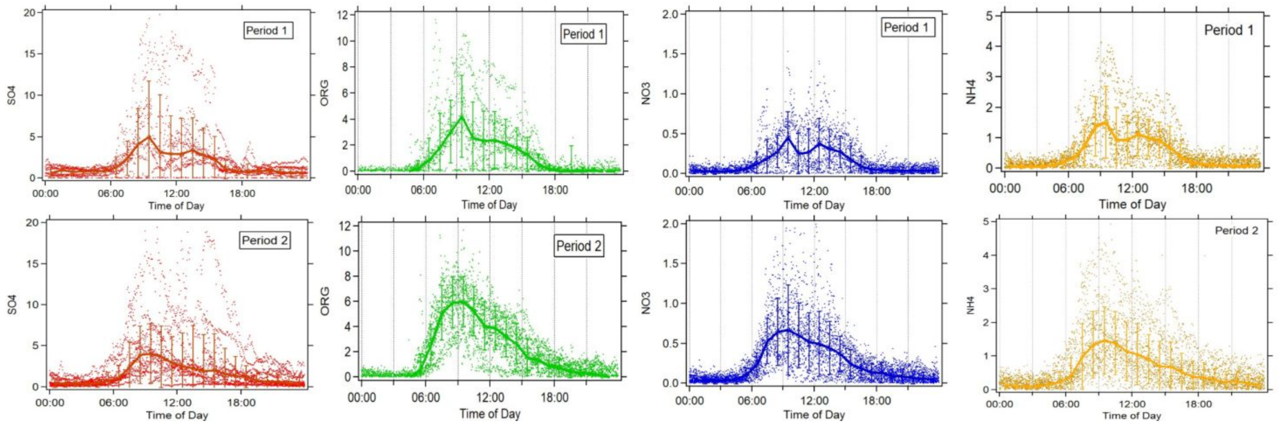

Figure S11. Diurnal profiles of ACSM chemical species (LT) observed in Period1 and Period2 of the field campaign.

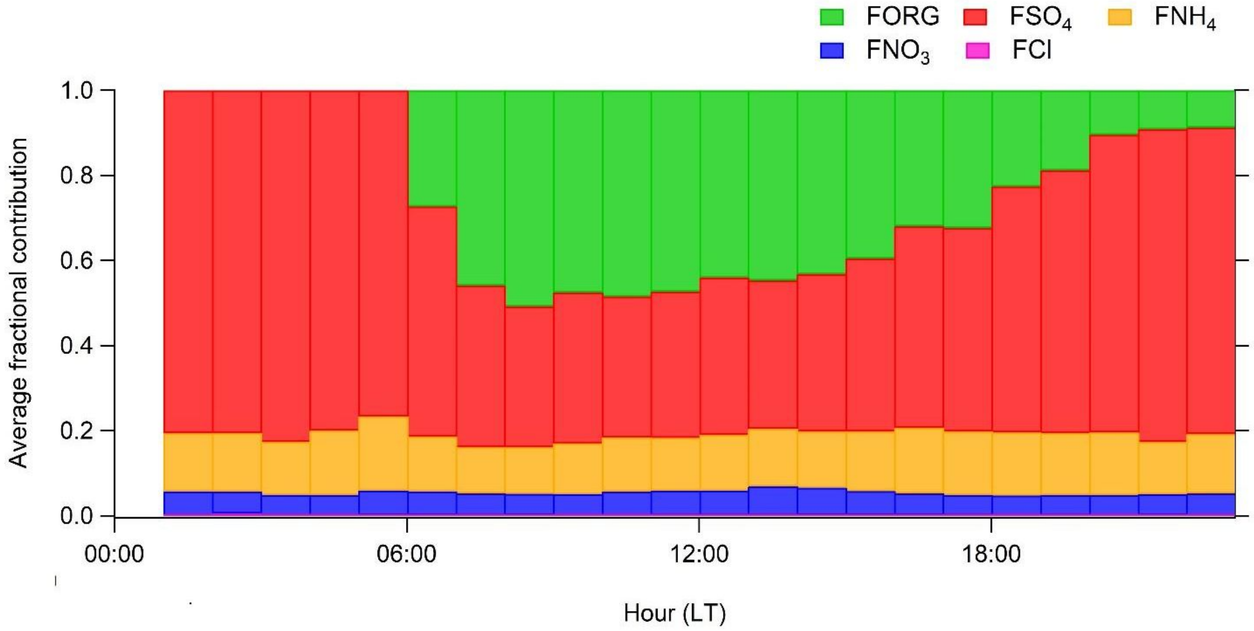

Figure S12. Relative diurnal contribution of NR-PM1 aerosols during the whole field campaign at MO.

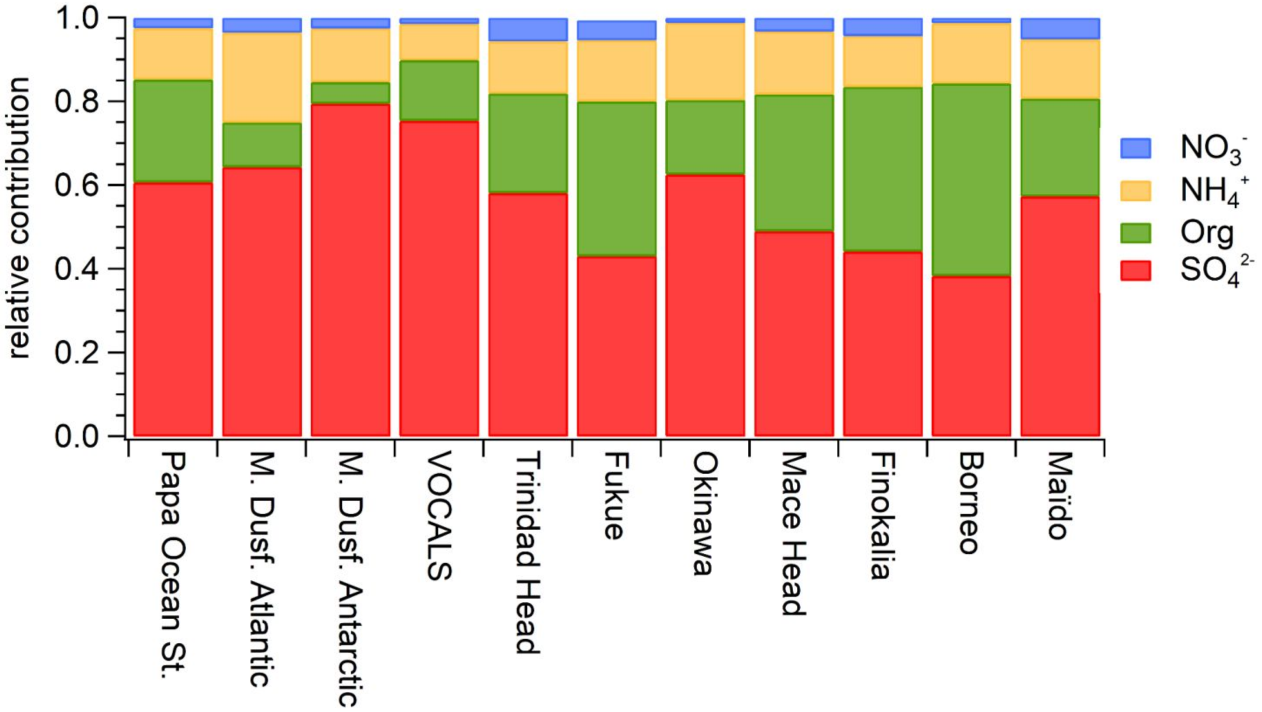

Figure S13. Average NR-PM_1 chemical contribution observed at MO (Réunion) and compared with the averages reported in the literature for other places worldwide, such as Fukue and Okinawa [5], Finokalia and Mace Head [6], Trinidad Head, VOCALS campaign (South Pacific), Ocean Station Papa, oceanic measurements obtained in the Atlantic Ocean and Antarctic by Marion Dusfrene (M. Dusf [7]) and in Borneo[11].

Section S5. Aerosol acidity

Acidity is estimated using the ion balance equations, which describe protons loading [H^+] (in μeq/m^3). The major difference between aerosol pH and the proton loading method is that pH is the H^+ concentration per liquid water volume. In contrast, aerosol proton loading is the H^+ concentration per unit volume of air. Aerosol pH is the parameter of interest for atmospheric phenomena, but the proton loading is often used instead as a simpler non-quantitative surrogate for pH [8].

$$[H^+] = [anions] - [cations] = \frac{Cl^-}{35.5} + \frac{NO_3^-}{62} + \frac{2*SO_4^{2-}}{48} - \frac{Na^+}{23} - \frac{NH_4^+}{18} - \frac{K^+}{39} - \frac{2*Mg^{2+}}{12} - \frac{2*Ca^{2+}}{20}$$

Relevant Pearson's correlation values were achieved in this study between [H^+] and malonic acid (R=0.4, p-value=0.02) and succinic acid (R=0.52, p-value=0.002). In contrast, oxalic acid did not show a significant correlation (R=0.28, p-value=0.12), suggesting preferential oxalic acid production in the aqueous phase. Equivalent NH_4^+/ (NO_3^- + SO_4^{2-}) ratios were also calculated (figure 5). A ratio of 1 would indicate that HNO_3 and H_2SO_4 were completely neutralized by NH_4^+ [9]. At our site, the average ratio is 0.24, indicating strong SO_4^{2-} concentrations prevalence over other species (From 2nd to 4th April in particular). These results suggest that

acidity may enhance the uptake of dicarboxylic acids, gaseous precursors, onto aerosols, as also reported by Feng et al.,[10].

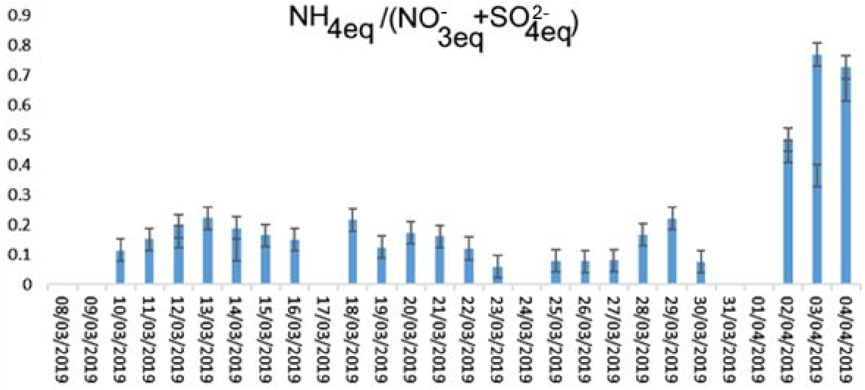

Figure S14. Equivalent $[NH_4^+]/(NO_3^-+SO_4^{2-})$ time series

Section S6. Reactive gas and volatile organic compounds measurements

In addition, the average NO_2 emissions during this study were low (0.020 ± 0.067 ppbv) in comparison to continental sites (0.432 ppbv to 2.980 ppbv at the Puy de Dôme station). CO mixing ratio (0.0300±0.0001 ppbv) was also much below the 80 ppbv maximum thresholds expected for remote mountain sites [4]. This combination of measurements suggests that minimal anthropogenic emissions influenced the aerosol particles measured at the site.

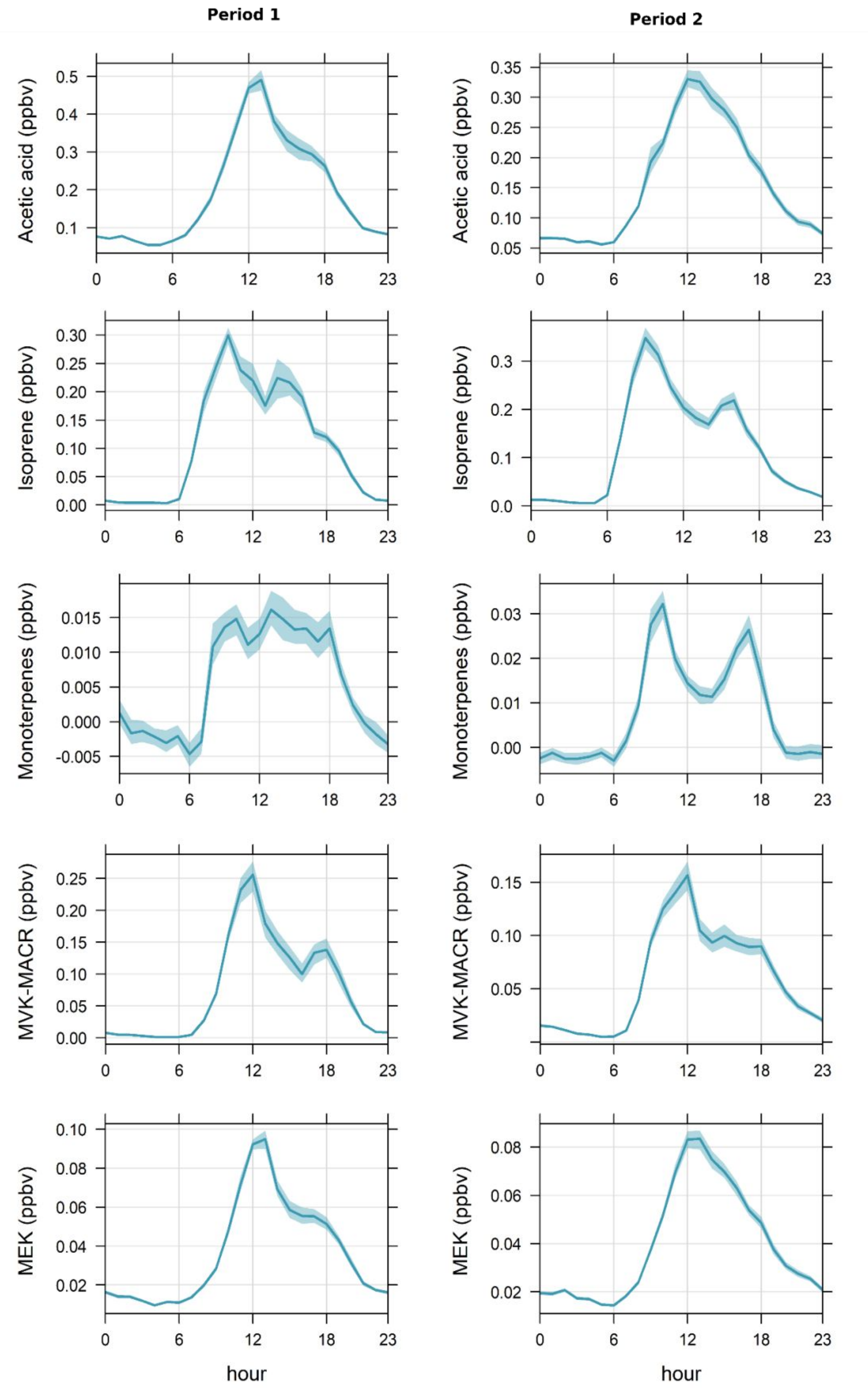

Figure S15. Selected VOCs average diurnal profiles (LT) during the first (8th March – 21st March, left) and second (22nd March – 4th April, right) periods of the BIO-MAIDO campaign. The bold lines represent average values, and surrounding light shaded areas represent confidence intervals at 95%.

Table S2. C5H6O+ (m/z 82) over Org at MO (Maido) compared with literature values [11]

	Borneo ensemble	Borneo PMF factor	Chen et al. Amazonia	European Boreal	North American temperate	**Maïdo**
$C_5H_6O^+$/ORG	1.24±0.01%	3.82%	0.68±0.01%	0.48±0.01%	0.40±0.01%	**1.19±0.01 %**

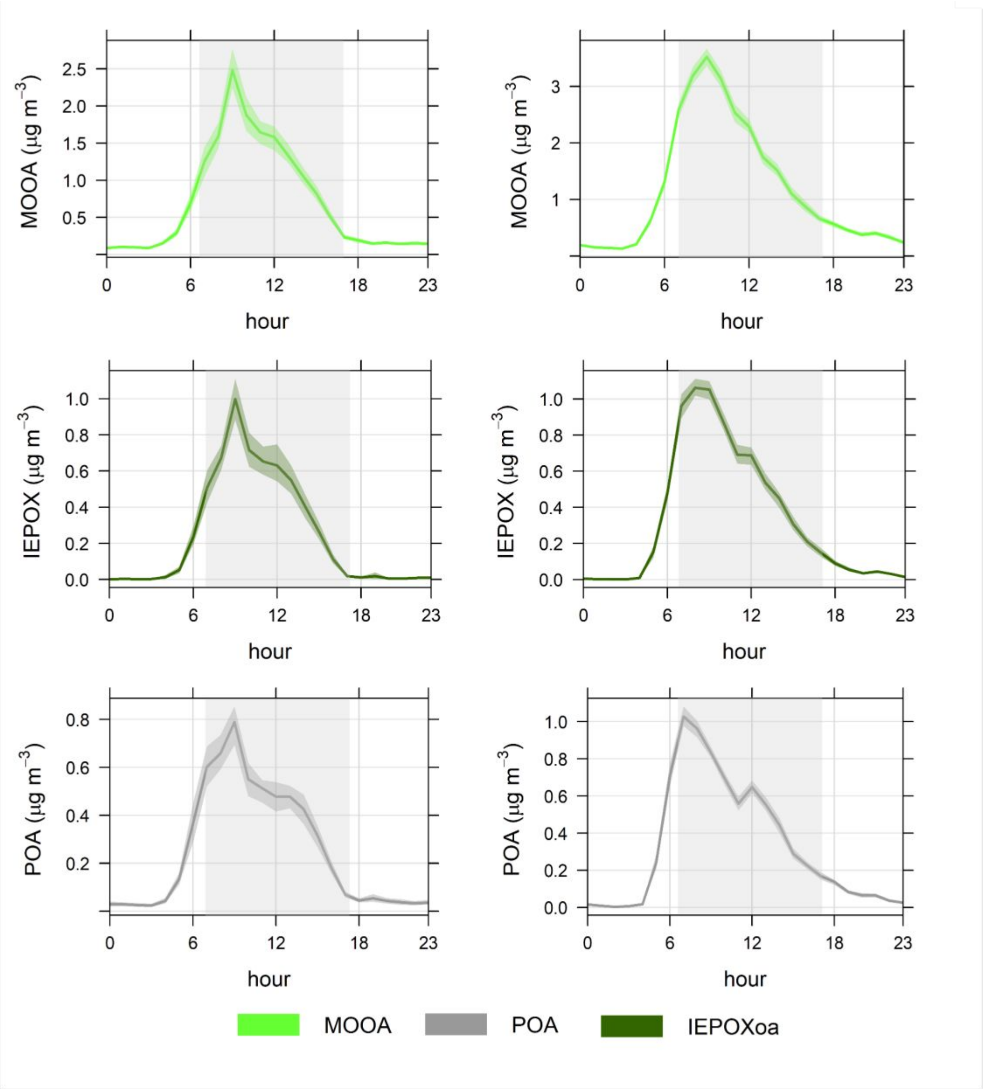

Figure S16. PMF factors diurnal variability (LT), MOOA (green), IEPOXOA (dark green), and POA (grey), separated into two sampling periods, on the Period 1 (left panels): from 13th March to 21st March (average of 9 days) and on the Period 2 (right panels): from 22nd March to 4th April (average of 14 days). Shaded areas represent hours with solar radiation.

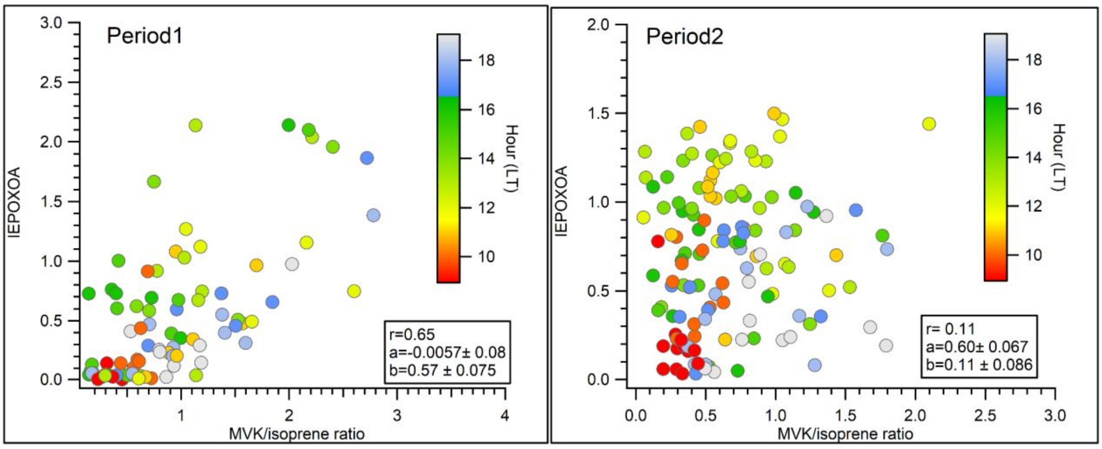

Figure S17. IEPOX vs. biogenic age (MVK MACR ISOPOOH/isoprene) colored by sampling date

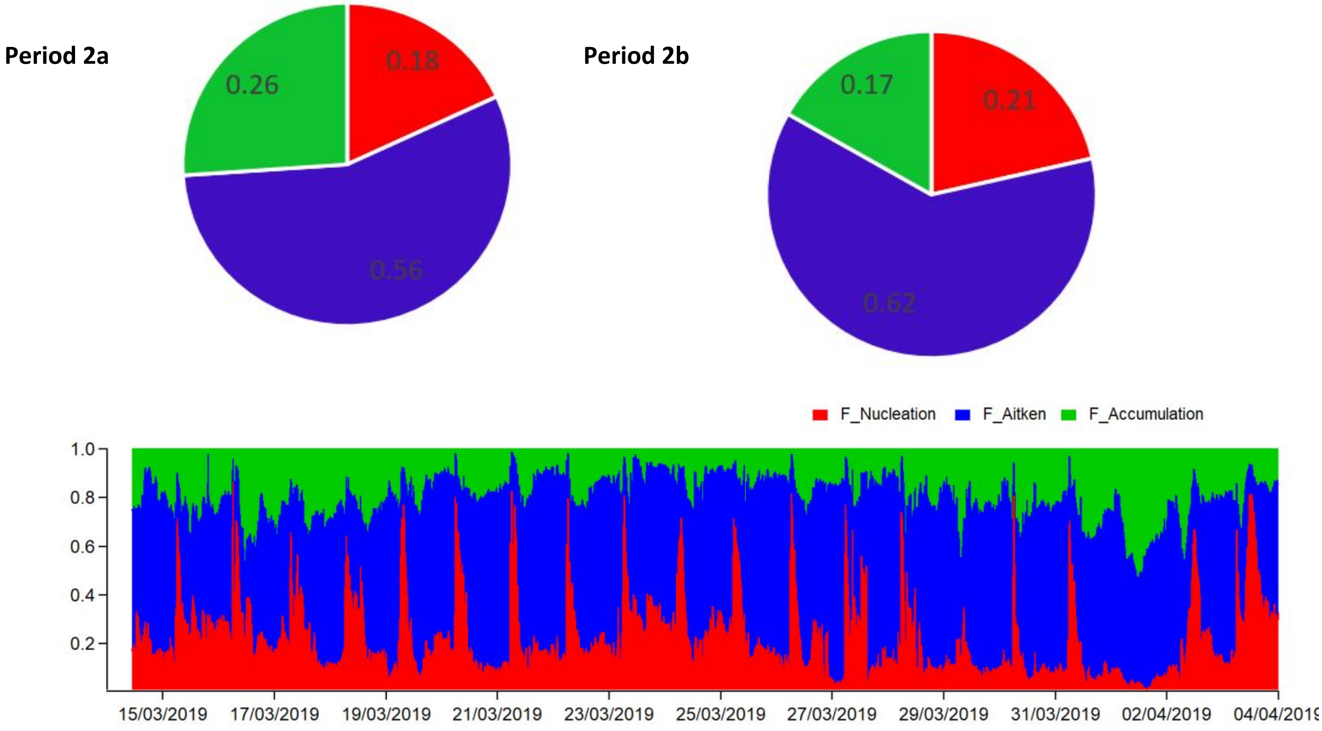

Figure S18. Aerosols size distribution time series observed during the whole field campaign at MO (bottom), and relative contribution of nucleation, aitken and accumulation fraction during Period 1 and Period 2 (top).

References

(1) Zhang, Q.; Jimenez, J. L.; Worsnop, D. R.; Canagaratna, M. A Case Study of Urban Particle Acidity and Its Influence on Secondary Organic Aerosol. *Environ. Sci. Technol.* **2007**, *41* (9), 3213–3219. https://doi.org/10.1021/es061812j.

(2) Coe, H.; Allan, J. D.; Alfarra, M. R.; Bower, K. N.; Flynn, M. J.; McFiggans, G. B.; Topping, D. O.; Williams, P. I.; O'Dowd, C. D.; Dall'Osto, M.; Beddows, D. C. S.; Harrison, R. M. Chemical and Physical Characteristics

of Aerosol Particles at a Remote Coastal Location, Mace Head, Ireland, during NAMBLEX. *Atmos. Chem. Phys.* **2006**, *6* (11), 3289–3301. https://doi.org/10.5194/acp-6-3289-2006.

(3) Takami, A.; Miyoshi, T.; Shimono, A.; Hatakeyama, S. Chemical Composition of Fine Aerosol Measured by AMS at Fukue Island, Japan during APEX Period. *Atmos. Environ.* **2005**, *39* (27), 4913–4924. https://doi.org/10.1016/j.atmosenv.2005.04.038.

(4) Zhou, S.; Collier, S.; Jaffe, D.; Zhang, Q. Free Tropospheric Aerosols at the Mt. Bachelor Observatory: More Oxidized and Higher Sulfate Content Compared to Boundary Layer Aerosols. *Atmos. Chem. Phys. Discuss.* **2018**, 1–25. https://doi.org/10.5194/acp-2018-821.

(5) Zhang, Q.; Jimenez, J. L.; Canagaratna, M. R.; Allan, J. D.; Coe, H.; Ulbrich, I.; Alfarra, M. R.; Takami, A.; Middlebrook, A. M.; Sun, Y. L.; Dzepina, K.; Dunlea, E.; Docherty, K.; DeCarlo, P. F.; Salcedo, D.; Onasch, T.; Jayne, J. T.; Miyoshi, T.; Shimono, A.; Hatakeyama, S.; Takegawa, N.; Kondo, Y.; Schneider, J.; Drewnick, F.; Borrmann, S.; Weimer, S.; Demerjian, K.; Williams, P.; Bower, K.; Bahreini, R.; Cottrell, L.; Griffin, R. J.; Rautiainen, J.; Sun, J. Y.; Zhang, Y. M.; Worsnop, D. R. Ubiquity and Dominance of Oxygenated Species in Organic Aerosols in Anthropogenically-Influenced Northern Hemisphere Midlatitudes. *Geophys. Res. Lett.* **2007**, *34* (13), n/a-n/a. https://doi.org/10.1029/2007GL029979.

(6) Bressi, M.; Cavalli, F.; Putaud, J. P.; Fröhlich, R.; Petit, J. E.; Aas, W.; Äijälä, M.; Alastuey, A.; Allan, J. D.; Aurela, M.; Berico, M.; Bougiatioti, A.; Bukowiecki, N.; Canonaco, F.; Crenn, V.; Dusanter, S.; Ehn, M.; Elsasser, M.; Flentje, H.; Graf, P.; Green, D. C.; Heikkinen, L.; Hermann, H.; Holzinger, R.; Hueglin, C.; Keernik, H.; Kiendler-Scharr, A.; Kubelová, L.; Lunder, C.; Maasikmets, M.; Makeš, O.; Malaguti, A.; Mihalopoulos, N.; Nicolas, J. B.; O'Dowd, C.; Ovadnevaite, J.; Petralia, E.; Poulain, L.; Priestman, M.; Riffault, V.; Ripoll, A.; Schlag, P.; Schwarz, J.; Sciare, J.; Slowik, J.; Sosedova, Y.; Stavroulas, I.; Teinemaa, E.; Via, M.; Vodička, P.; Williams, P. I.; Wiedensohler, A.; Young, D. E.; Zhang, S.; Favez, O.; Minguillón, M. C.; Prevot, A. S. H. A European Aerosol Phenomenology - 7: High-Time Resolution Chemical Characteristics of Submicron Particulate Matter across Europe. *Atmos. Environ. X* **2021**, *10*. https://doi.org/10.1016/j.aeaoa.2021.100108.

(7) Shank, L. M.; Howell, S.; Clarke, A. D.; Freitag, S.; Brekhovskikh, V.; Kapustin, V.; McNaughton, C.; Campos, T.; Wood, R. Organic Matter and Non-Refractory Aerosol over the Remote Southeast Pacific: Oceanic and Combustion Sources. *Atmos. Chem. Phys.* **2012**, *12* (1), 557–576. https://doi.org/10.5194/acp-12-557-2012.

(8) Hennigan, C. J.; Izumi, J.; Sullivan, A. P.; Weber, R. J.; Nenes, A. A Critical Evaluation of Proxy Methods Used to Estimate the Acidity of Atmospheric Particles. *Atmos. Chem. Phys.* **2015**, *15* (5), 2775–2790. https://doi.org/10.5194/acp-15-2775-2015.

(9) Kumar, P.; Yadav, S. Seasonal Variations in Water Soluble Inorganic Ions, OC and EC in PM10 and PM>10 Aerosols over Delhi: Influence of Sources and Meteorological Factors. *Aerosol Air Qual. Res.* **2016**, *16* (5), 1165–1178. https://doi.org/10.4209/aaqr.2015.07.0472.

(10) Feng, J. L.; Guo, Z. G.; Zhang, T. R.; Yao, X. H.; Chan, C. K.; Fang, M. Source and Formation of Secondary Particulate Matter in PM2.5 in Asian Continental Outflow. *J. Geophys. Res. Atmos.* **2012**, *117* (3), 1–11. https://doi.org/10.1029/2011JD016400.

(11) Robinson, N. H.; Hamilton, J. F.; Allan, J. D.; Langford, B.; Oram, D. E.; Chen, Q.; Docherty, K.; Farmer, D. K.; Jimenez, J. L.; Ward, M. W.; Hewitt, C. N.; Barley, M. H.; Jenkin, M. E.; Rickard, A. R.; Martin, S. T.; McFiggans, G.; Coe, H. Evidence for a Significant Proportion of Secondary Organic Aerosol from Isoprene above a Maritime Tropical Forest. *Atmos. Chem. Phys.* **2011**, *11* (3), 1039–1050. https://doi.org/10.5194/acp-11-1039-2011.

(12) Dominutti, P. A.; Renard, P.; Vaïtilingom, M.; Bianco, A.; Baray, J.-L.; Borbon, A.; Bourianne, T.; Burnet, F.; Colomb, A.; Delort, A.-M.; Duflot, V.; Houdier, S.; Jaffrezo, J.-L.; Joly, M.; Leremboure, M.; Metzger, J.-M.; Pichon, J.-M.; Ribeiro, M.; Rocco, M.; Tulet, P.; Vella, A.; Leriche, M.; Deguillaume, L. Insights into Tropical Cloud Chemistry in Réunion (Indian Ocean): Results from the BIO-MAÏDO Campaign. *Atmos. Chem. Phys.* **2022**, *22* (1), 505–533. https://doi.org/10.5194/acp-22-505-2022.

(13) Masson, V., Champeaux, J. L., Chauvin, F., Meriguet, C., & Lacaze, R. A global database of land surface parameters at 1-km resolution in meteorological and climate models. Journal of Climate, **2003**, 16(9), 1261–1282. https://doi.org/10.1175/1520-0442-16.9.1261

(14) Lac, C., Chaboureau, J. P., Masson, V., Pinty, J. P., Tulet, P., Escobar, J., et al. Overview of the meso-NH model version 5.4 and its applications. Geoscientific Model Development, **2018**, 11(5), 1929–1969. https://doi.org/10.5194/gmd-11-1929-2018

(15) Rocco, M., Baray, J.-L., Colomb, A., Borbon, A., Dominutti, P., Tulet, P., et al. High resolution dynamical analysis of volatile organic compounds (VOC) measurements during the BIO-MAÏDO field campaign (Réunion Island, Indian Ocean). Journal of Geophysical Research: Atmospheres, **2022**, 127, e2021JD035570. https://doi-org.insu.bib.cnrs.fr/10.1029/2021JD035570
